# FtMt reduces oxidative stress-induced trophoblast cell dysfunction via the HIF-1α/VEGF signaling pathway

**DOI:** 10.1186/s12884-023-05448-1

**Published:** 2023-03-01

**Authors:** Xia Xu, Xu Ye, Mengwei Zhu, Qiuyu Zhang, Xiuli Li, Jianying Yan

**Affiliations:** Fujian Maternity and Child Health Hospital, College of Clinical Medicine for Obstetrics & Gynecology and Pediatrics, Fujian Medical University, Fuzhou, 350001 Fujian China

**Keywords:** Preeclampsia, Mitochondrial ferritin, HIF-1α/VEGF signaling pathway

## Abstract

**Background:**

Preeclampsia (PE) is a complication of pregnancy that causes long-term adverse outcomes for the mother and fetus and may even lead to death. Oxidative stress caused by the imbalance of oxidants and antioxidants in the placenta has been considered as one of the key mechanisms of preeclampsia (together with inflammation, etc.), in which the placental mitochondria play an important role. The expression of hypoxia-inducible factor-1 (HIF-1α) and vascular endothelial growth factor (VEGF) is known to be increased in patients with PE. Mitochondrial ferritin (FtMt) is known to protect the mitochondria from oxidative stress, although its specific role in PE remains unclear.

**Methods:**

We used qRT-PCR and western blotting to detect the expression levels of FtMt, HIF-1α, and VEGF in placental tissues from patients with PE. Human chorionic trophoblast cells were also administered with hypoxia treatment, followed by the detection of cell proliferation, invasion and angiogenic capacity by CCK8, Transwell, and endothelial cell angiogenesis assays; we also detected the expression of HIF-1α and VEGF in these cells. Finally, overexpression or inhibitory FtMt lentiviral vectors, along with negative control vectors, were constructed and transfected into hypoxia-treated human chorionic trophoblast cells; this was followed by analyses of cell function.

**Results:**

The expression levels of FtMt, HIF-1α and VEGF in the PE group were higher than those in the control group (*P* < 0.05). Following hypoxia, there was an increase in the expression levels of HIF-1α and VEGF protein in trophoblast cells. There was also an increase in invasion ability and vascular formation ability along with a reduction in cell proliferation ability. These effects were reversed by transfecting cells with the knockout FtMt lentivirus vector. The differences were statistically significant.

**Conclusion:**

Analyses showed that FtMt plays a key role in the vascular regulation of PE trophoblast cells after hypoxia possibly acting via the HIF-1α/VEGF signaling pathway. These results provide us an enhanced understanding of the pathogenesis of PE and suggest that the HIF-1α/VEGF signaling pathway represents a new target for the treatment of PE.

**Supplementary Information:**

The online version contains supplementary material available at 10.1186/s12884-023-05448-1.

## Background

Preeclampsia (PE) is a clinical syndrome of pregnancy that seriously threatens the health of both mothers and infants [1]. Currently, the global incidence of PE ranges from 3 to 5%, and represents one of the main causes of maternal mortality [2–4]. PE is a gestational idiopathic disease that is characterized by a systolic blood pressure of 140 mmHg or higher, a diastolic blood pressure of 90 mmHg or higher, or both systolic and diastolic blood pressure above ≥ 140/90 mmHg, measured twice with a four-hour interval, with proteinuria in 24 h urine ≥ 300 mg or protein/creatinine ratio ≥ 0.3 [5, 6]. As the main cause of maternal and perinatal morbidity and mortality, PE involves basic pathophysiological changes that include vascular endothelial cell damage and systemic small artery spasm. This reduces perfusion in each body system, thus leading to multiple organ damage. Multiple clinical studies of women with preeclampsia show an increased risk of developing cardiovascular diseases later in life. Additional adverse outcomes, such as the increased risk of renal disease, metabolic disorders, and death, have also been reported [7]. Due to the risk and importance of PE, it is very important to diagnose and treat PE in a timely manner.

The development of preeclampsia may cause placental hypoxia which is characterized by the up-regulated expression of hypoxia-induced transcription factor (HIF) [8] and poor angiogenesis in the placenta. Placental hypoxia is also the pathological basis for several clinical manifestations, including hypertension, proteinuria and fetal growth restriction [9]. Poor placental angiogenesis may be due to the dysregulation of growth factors such as vascular endothelial growth factor (VEGF) and related intracellular signaling pathways [10–12].

Oxidative stress injury in the placental mitochondria is one of the mechanisms known to be involved in the pathogenesis of preeclampsia. Oxidative stress is caused by reduced levels of placental perfusion due to abnormal stenosis of the spiral arteries, thus resulting in ischemia and reperfusion injury [13]. This oxidative stress is caused by an imbalance between the production and expenditure of oxidants. Mitochondria provide 90% of cellular metabolic energy via aerobic respiration [14]. Mitochondria dysfunctional becomes the main focus of oxidative stress both in and outside the cell.

Mitochondrial ferritin (FtMt) is a ferritin storage protein that exhibits ferrous oxidase activity and is expressed in the mitochondria [15]. The overexpression of FtMt in cultured cells can reduce the levels of oxidative stress, and therefore oxidative damage, by reducing the amount of iron in the intracellular free iron pool [16, 17]. Studies have found that FtMt expression is enhanced under oxidative stress conditions, where FtMt protects cells against the oxidative stress [18]. In addition, the study of FtMt indicates that it participates in the protection against oxidative damage, particularly in cells with high oxidative activity [19]. However, the role of FtMt in the pathogenesis of PE is rarely studied, and it remains unclear. Whether FtMt reduces oxidative stress injury in PE by increasing the expression of HIF-1α/VEGF remains unclear.

In this study, we investigated the role of FtMt in the vascular regulation of PE trophoblast cells after hypoxia via the HIF-1α/VEGF signaling pathway. Our intention was to provide an important theoretical basis for an enhanced understanding of the pathogenesis of PE and to identify new targets for the treatment of PE.

## Methods

### Sample source

10 cases of PE served in a study group and 10 cases of normal late pregnancy were allocated to a control group in which women underwent cesarean section due to a history of cesarean section, abnormal birth canal, abnormal fetal position, or social factors but without pregnancy complications. All clinical samples were collected from Fujian Maternity and Child Health Hospital.

Preeclampsia and the severity of preeclampsia were diagnosed using standardized criteria suggested by the American College of Obstetricians and Gynecologists [20]. PE is characterized by systolic blood pressure ≥ 140 mmHg or diastolic blood pressure ≥ 90 mmHg appeared for the first time after 20 weeks of gestation, and proteinuria was defined as urinary protein > 0.3 g/24 h. Mild PE patients had systolic blood pressure ≥ 140 mm Hg, diastolic blood pressure ≥ 90 mmHg on 2 occasions separated by 6 h, and proteinuria > 0.3 g/24 h. Severe PE can be diagnosed if at least one of the following criteria is met: patients on bed rest had systolic blood pressure ≥ 160 mmHg or diastolic blood pressure ≥ 110 mmHg twice, 6 h apart; Proteinuria ≥ 5 g/24 h; Or twice 4 h interval proteinuria; Oliguria, 24-h urine volume < 500 ml; Brain or visual impairment; Pulmonary edema or cyanosis; Epigastric or right upper abdominal pain; Impaired liver function; Thrombocytopenia; Fetal growth restriction. According to gestational age at the time of diagnosis, gestational age less than 34 weeks was early onset PE and gestational age greater than or equal to 34 weeks was late onset PE. Study population included all PE patients with early and late onset, mild and severe. Criteria for exclusion from the study were patient failure, premature rupture of membranes, chorioamnitis, diabetes, autoimmune diseases, and severe heart, kidney, and liver disease prior to pregnancy. All pregnant women in the PE group were delivered by cesarean section because of surgical indications. The pregnant women in the two groups were not in labor and the fetal membranes were not ruptured during cesarean section. Patients with other pregnancy complications or medical/surgical complications were excluded from the study.

Human placental tissue was collected during cesarean section. Once the placenta had been delivered, we immediately removed a portion of placental villous tissue (1.0 cm^3^) without decidua from the root of the umbilical cord. We ensured that this procedure was carried out under aseptic conditions and made sure that tissue was only removed from areas on the placenta that did not show bleeding, infarction, or calcification. After the placental tissue samples were collected, the residual blood was washed with normal saline, and 5 times the volume of RNAlater was added to preserve at -80 degrees. Placenta samples were 1–10 in the control group (No: N1-N10) and 1–10 in the experimental group (No: P1-P10). The acquisition of cases included in the study was conducted in accordance with ethical procedures and approved by the Ethics Committee of Fujian Maternity and Child Health Hospital (Reference: 2021KLD661). All samples were collected with the consent of the patients themselves or their families and the informed consent signed in advance.

### Reverse transcription and quantitative PCR

Total RNA was extracted from placental tissue by RNAiso Plus (TaKaRa Bio, Otsu, Japan) according to the instructions. The concentration of RNA extracted from each sample was accurately measured by Q5000 UV–Vis spectrophotometer (Quawell, USA). The value of OD260: OD280 was used to evaluate the quality of RNA extraction. RNA integrity was detected by 2% agarose gel electrophoresis. Reverse transcription was performed according to the instructions of the NovoScript® 1ST Strand cDNA Synthesis SuperMix reverse transcription kit. The internal reference gene was GAPDH, and all primers were synthesized by Fuzhou Shangya Biological Company. The sequences were GAPDH, F: GAGAGACCCTCACTGCTG, R: TGGTACATGACAAGGTGCGG. HIF-1α, F: CCGAATTGATGGGATATGAG, R: TCATGATGAGTTGGTCAGATG; VEGF, F: GAGCCTTGCCTTGCTGCTCTAC, R: CACCAGGGTCTCGATTGGATG; FtMt, F: CAGGTATTTCCTTCACCAGTCC, R: GTTCCGGCTTCTTGATGTCC. After the specificity of the primers was verified by common PCR, qRT-PCR was performed using NovoStart® SYBR qPCR SuperMix Plus (NovoProtein) kit. Each reaction is repeated three times. The expression level of F = 2^−△△CT^ gene was analyzed by relative quantitative method.

Total RNA was extracted from cells by NucleoZol method according to the instructions, and then reverse transcription. With β-actin as internal reference, the expression levels of HIF1a, VEGF and FtMt were detected by fluorescence quantitative PCR (LineGene 9600 series, Bori, Hangzhou). Primer sequences are shown in Table1 below.

**Table 1:** Primer sequences

Gene	Forward primer sequence (5’-3’)	Reverse primer sequence (5’-3’)
HIF-1α	GCAGCAACGACACAGAAACT	TGCAGGGTCAGCACTACTTC
VEGF	ATCGAGTACATCTTCAAGCCAT	GTGAGGTTTGATCCGCATAATC
FtMt	TGCCATGGAGTGTGCTCTAC	AATCGCACAAATGGGGGTCA
β-Actin	TGACGTGGACATCCGCAAAG	CTGGAAGGTGGACAGCGAGG

### Cell culture and hypoxia treatment

The trophoblast cell line HTR8/SVneo were purchased from Procell Life Science & Technology Co., Ltd. (Wuhan, China.) and cultured in DMEM-H medium (Gibco, USA) containing, 100 U/ml Penicillin–Streptomycin (Gibco, USA), and 10% fetal bovine serum (FBS, Gibco, USA). Cells which cultured at 37℃ and 5%CO2 conditions were adopted as normal control. And cells in the hypoxia group were placed under hypoxia (1% O2) in anoxic box, which can freely adjust oxygen concentration and flow meter detection, closed and cultured at 37℃ and 5%CO2. Cells were collected 48 h later for subsequent experiments.

### Generation of stably transfected cell lines

shRNA-FtMt1, shRNA-FtMt2, shRNA-FtMt3, shRNA-NC, oe-FtMt and oe-NC viruses were purchased from General Biosystems (Anhui, China) Co., Ltd. shRNA-FtMt1, shRNA-FtMt2 and shRNA-FtMt3 were three different lentiviral vector sequences for FtMt interference and we chose the one with the highest inhibitory efficiency for subsequent experiments. oe-FtMt was the lentiviral vector for FtMt overexpression. The sh-FtMt1 sequences were: 5-GATCCGGACATCAAGAAGCCGGAACATTCAAGAGATGTTCCGGCTTCTTGATGTCCTTTTTG-3 and 5-AATTCAAAAAGGACATCAAGAAGCCGGAACATCTCTTGAATGTTCCGGCTTCTTGATGTCCG-3; The sh-FtMt2 sequences were: 5-GATCCGCGATTTCCTGGAAACCTACTTTCAAGAGAAGTAGGTTTCCAGGAAATCGCTTTTTG-3 and 5-AATTCAAAAAGCGATTTCCTGGAAACCTACTTCTCTTGAAAGTAGGTTTCCAGGAAATCGCG-3; The sh-FtMt3 sequences were: 5-GATCCGCAGGTGAAGTCTATCAAAGATTCAAGAGATCTTTGATAGACTTCACCTGCTTTTTG-3 and 5-AATTCAAAAAGCAGGTGAAGTCTATCAAAGATCTCTTGAATCTTTGATAGACTTCACCTGCG-3; The oe-FtMt sequences were: 5-ATGCTGTCCTGCTTCAGGCTCCTCTCCAGGCACATCAGCCCTTCGCTGGCGTCTCTGCGCCCGGTGCGCTGCTGCTTCGCGCTCCCGCTGCGTTGGGCCCCGGGGCGCCCCTTGGACCCCAGGCAGATCGCCCCCCGCCGCCCCCTGGCCGCAGCCGCCTCCTCCCGGGACCCTACCGGGCCCGCCGCCGGCCCCTCTCGGGTGCGCCAGAACTTCCACCCCGACTCCGAGGCTGCCATCAACCGCCAGATCAACCTCGAGCTCTATGCGTCCTACGTGTACTTGTCCATGGCCTATTACTTCTCCCGGGATGACGTGGCCTTGAACAACTTCTCCAGGTATTTCCTTCACCAGTCCCGGGAGGAGACCGAGCACGCGGAGAAGCTGATGAGGCTGCAGAACCAGCGAGGAGGCCGGATCCGCCTGCAGGACATCAAGAAGCCGGAACAGGACGACTGGGAAAGCGGGCTGCATGCCATGGAGTGTGCTCTACTCTTGGAAAAGAACGTGAACCAGTCGTTGCTGGAATTGCACGCTCTAGCCTCAGATAAAGGTGACCCCCATTTGTGCGATTTCCTGGAAACCTACTACCTGAATGAGCAGGTGAAGTCTATCAAAGAACTAGGTGACCACGTGCACAACTTAGTGAAGATGGGGGCCCCGGATGCTGGCCTGGCGGAGTACCTTTTTGACACACATACCCTTGGAAATGAAAACAAGCAGAACTAA-3. shRNA-NC and oe-NC lentivirus vector were pLVZG-U6-ZsGreen1-Puro and pLVZG-CMV-copGFP-Puro which were devoid of a functional sequence. The transfection of HTR8/SVneo cells with either shRNA-FtMt1, shRNA-FtMt2, shRNA-FtMt3, shRNA-NC, oe-FtMt or oe-NC by utilizing the Lipofectamine 2000 Transfection Reagent (Invitrigen, USA) according to the manufacturer’s instructions.

Logarithmic growing cells were transferred into 6-well plates and cultured overnight at 37℃ and then cells were infected with virus for 6 h. Replenish 1 ml complete culture medium. After 24 h of infection, the virus-containing medium was sucked out and 2 ml fresh medium was added to continue culture. Fluorescence expression was observed under a fluorescence microscope (Nikon ECLIPSE Ts2R-FL) after infection 48–72 h. The cells were used for hypoxia treatment and functional analysis.

### Western blot analysis

The cells were collected and RIPA lysis buffer (Meilun Biotechnology Co., Ltd., Suzhou, China) containing PMSF (1 mM) (Meilun Biotechnology Co., Ltd., Suzhou, China) was added for protein extraction. Total protein concentration of samples was detected by BCA protein assay. After diluting the protein sample to the same concentration with RIPA lysis solution, added 5 × loading buffer, boiled in 100 °C water bath for 5 min, and store at -80 °C after cooling on ice. 30 μg total protein samples were taken from each group, separated by SDS-PAGE gels electrophoresis and transferred to PVDF membranes. After sealing with 5% bovine serum albumin (Albumin from bovine serum, BSA), the membranes were incubated with primary antibodies anti-VEGF (1:2000, 66,828–1-IG; proteintech), anti-HIF-1α (1:2000, 66,730–1-IG; proteintech), or anti-GAPDH (1:2000, 60,004–1-IG; proteintech). Following incubation with the secondary antibody solution of HRP Goat Anti Mouse IgG (H + L) (1:5000, proteintech). The PVDF membranes were color-coded by chemiluminescence method and luminescence detection by gel imaging system Versa DocTM Imaging System to collect strip images. GAPHD was used as internal reference to calculate the relative expression levels of each target protein by Image Lab analysis software (National Institutes of Health).

### Cell proliferation detection

The viability of HTR-8/SVneo cells was detected using a CCK-8 kit (Dojindo, Japan). After 48 h of hypoxia, the cells were inoculated into 96-well plates, and 10 μl 10% CCK-8 solution was added to each well at 0, 24, 48 and 72 h, respectively, and cultured for 1 ~ 2 h. The absorbance value of each hole was detected at OD450nm.

### Detection of cell invasion ability

The Martrigel matrix glue was placed in the refrigerator at 4 ℃ until completely melted. The standard Martrigel glue was diluted 1:8 on ice in serum-free medium. The 50 μL diluted Martrigel glue was placed into the chamber by Transwell, air-dried at 4 ℃, and solidified at 37 ℃. Place in 24-well plate. 100μL of T24 cells in each group (1 × 105 cells /mL) were inoculated into the upper chamber of Transwell, 600 µl medium containing 20% FBS was added into the lower chamber, and cultured in a 37℃ incubator for 24 h. The upper chamber cells were wiped with cotton swabs. The cells were fixed with 600 μl 4% paraformaldehyde in the lower chamber for 30 min, then the paraformaldehyde was removed and the cells that did not pass through the membrane were wiped with cotton swabs. The lower chamber was stained with 600 μl 0.1% crystal violet for 15 min. The cells were washed with PBS twice. Each group was randomly selected to take pictures under a microscope, observe and count cells.

### Inner tube formation experiment

Cells were added into a 96-well plate coated with Matrigel in 3 × 10^4^/well, 100 μl for each well in the complete medium. The optimum tube forming time was selected within 8 h of culture at 37℃ (observed at intervals). Under an inverted microscope, the formation effect of the inner tube was observed at 100 × magnification, and the field of vision was randomly selected for photographing and preservation.

### Statistical analysis

SPSS17.0 statistical software was used for data processing. The measurement data were expressed as mean ± standard deviation (SD), t-test was used for comparison between groups, and χ2 test was used for comparison of count data. Differences among multiple groups were analyzed by one way analysis of variance. Data distribution was tested by Kolmogorov–Smirnov test to state the type of statistical analysis. *P* values < 0.05 were considered to be statistically significant.

## Results

In this study, we investigated the role of FtMt in the vascular regulation of PE trophoblast cells after hypoxia via the HIF-1α/VEGF signaling pathway. We studied the expression levels of HIF-1α, FtMt and VEGF in placental tissue of PE patients and explored the function of trophoblast cells by changing oxygen conditions. In addition, we demonstrated that FtMt affects trophoblast function under hypoxia through HIF-1α/VEGF signaling pathway.

### Clinical characteristics

The clinical data of all the study’s participants were no statically significant in Maternal age and body mass index (BMI) (Table 2). The gestational age in the PE group was significantly lower than in the control group (*P* < 0.05). Systolic blood pressure and Diastolic blood pressure in the PE group was significantly higher than in the control group (*P* < 0.05) (Supplementary Table).

**Table 2:** Clinical characteristics of study population

Parameters	Normal (*n* = 10)	Preeclampsia (*n* = 10)	*p* value Preeclampsia vs. Normal
Maternal age (year)	30.2 ± 1.2	31.9 ± 1.5	*p* = 0.359
BMI pre-pregnancy (kg/m2)	25.8 ± 0.4	28.6 ± 1.3	*p* = 0.093
Gestational age (week)	38 ± 0.3	33 ± 0.6	*p* < 0.05
Systolic blood pressure at delivery (mmHg)	117 ± 1.9	152 ± 3.7	*p* < 0.05
Diastolic blood pressure at delivery (mmHg)	72 ± 1.6	100 ± 2.6	*p* < 0.05
Newborn gender			*p* = *0.371*
Male	6	4	
Female	4	6	

Data are presented as mean ± SD or percentage

*P* value < 0.05 was considered as significant difference

BMI, body mass index in pregnancy (kg/m^2^)

Descriptive statistics includes testing differences between All PE and normal pregnancy groups using Chi-Square tests and t-tests

### HIF-1α, FtMt and VEGF mRNA expression in placental tissues

**Fig. 1:**
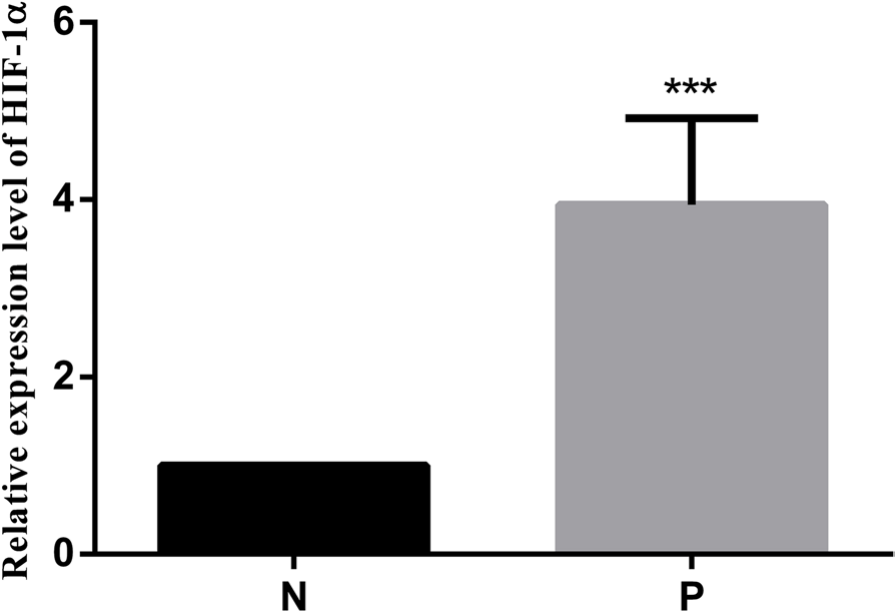
Expression levels of HIF-1α in placental tissues. mRNA expression of HIF-1α in placental tissues detected by RT-qPCR. Data are depicted as means ± SEM (*n* = 10/group). Statistical significance assessed by t tests. N: Normal group. P: PE group. ***P < 0.001

**Fig. 2:**
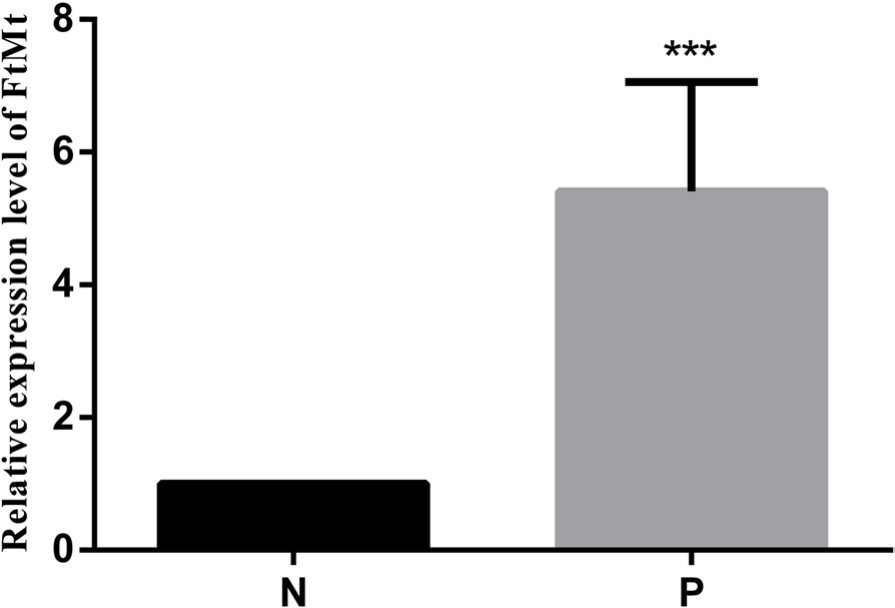
Expression levels of FtMt in placental tissues. mRNA expression of FtMt in placental tissues detected by RT-qPCR. Data are depicted as means ± SEM (*n* = 10/group). Statistical significance assessed by t tests. N: Normal group. P: PE group. ***P < 0.001

**Fig. 3:**
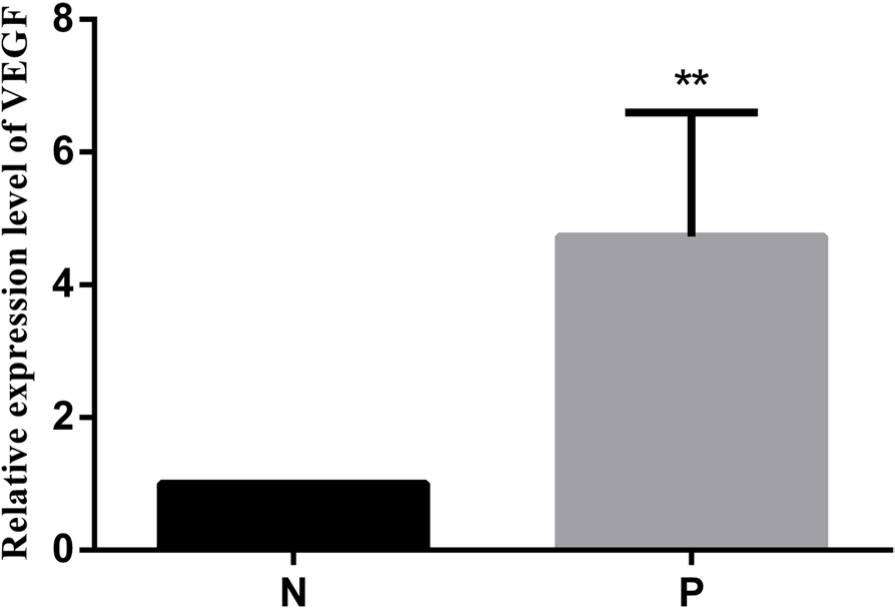
Expression levels of VEGF in placental tissues. mRNA expression of VEGF in placental tissues detected by RT-qPCR. Data are depicted as means ± SEM (*n* = 10/group). Statistical significance assessed by t tests. N: Normal group. P: PE group. **P < 0.01

The relative expression levels of HIF-1α, FtMt and VEGF in placental tissue samples were determined by qRT-PCR. We found that the mRNA expression levels of HIF-1α and FtMt in the PE group were significantly higher than those in the normal control group (*P* < 0.05; Figs. 1 and 2). Furthermore, the VEGF expression of PE patients were also increased (*P* < 0.05; Fig. 3).

### The proliferation and invasion ability of trophoblast cells and the expression of HIF-1α and VEGF following hypoxia

#### Morphological changes of HTR8 cells after hypoxia

**Fig. 4:**
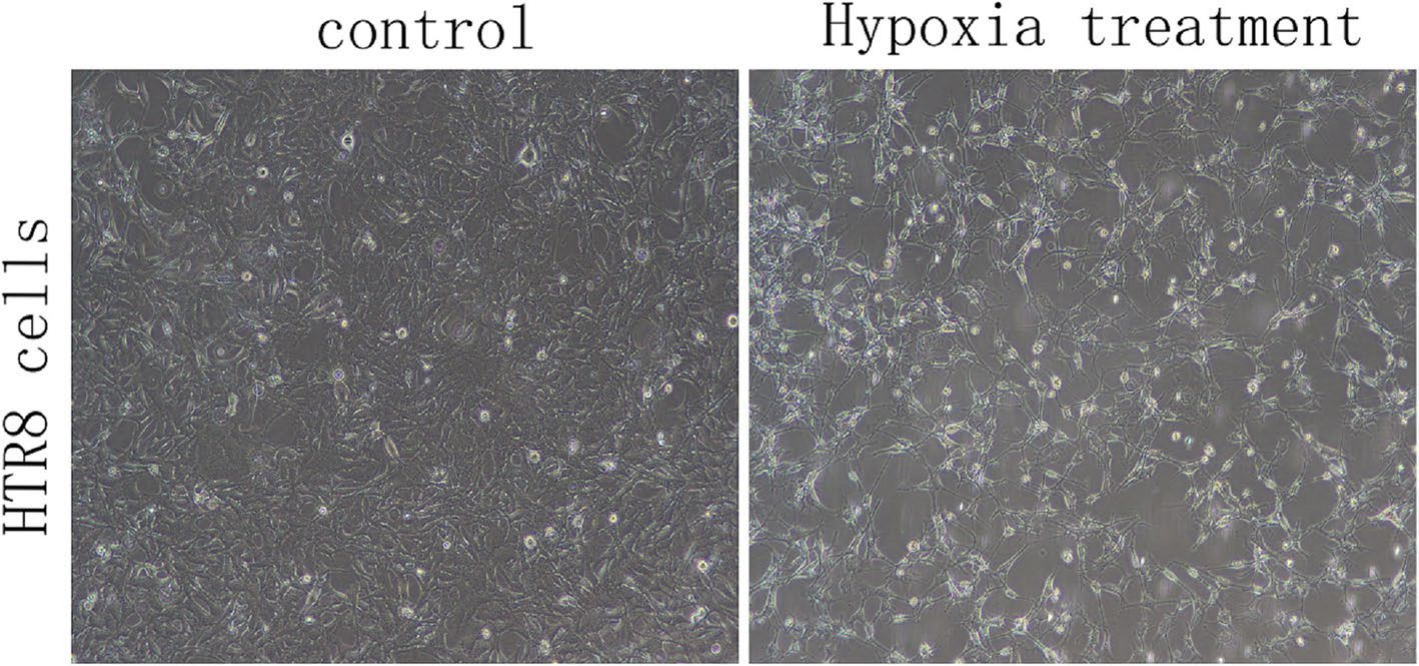
Morphological changes of HTR8 cells in hypoxia group and normal group. (100 ×) (*n* = 1 per group)

Human chorionic trophoblast cells were cultured in a hypoxia box at 37℃ and 5%CO_2_. The hypoxia treatment group represented the experimental group while the control group (normal) was cultured at 37℃ and 5%CO_2_. During the logarithmic growth phase, the two groups of cells were inoculated on 6-well plates and cell morphology was observed by inverted phase contrast microscopy (Fig. 4). In the control group, HTR-8 /SVneo cells showed normal morphological structure and clear cell contours. In the hypoxia treatment group, the cell morphology had changed significantly, including cell shrinkage and a reduction in both gap width and volume. Some cells showed flake necrosis and shedding; there was a reduction in the number of surviving cells.

#### HIF-1α and VEGF expression in oxidative stress-induced trophoblast cell

**Fig. 5:**
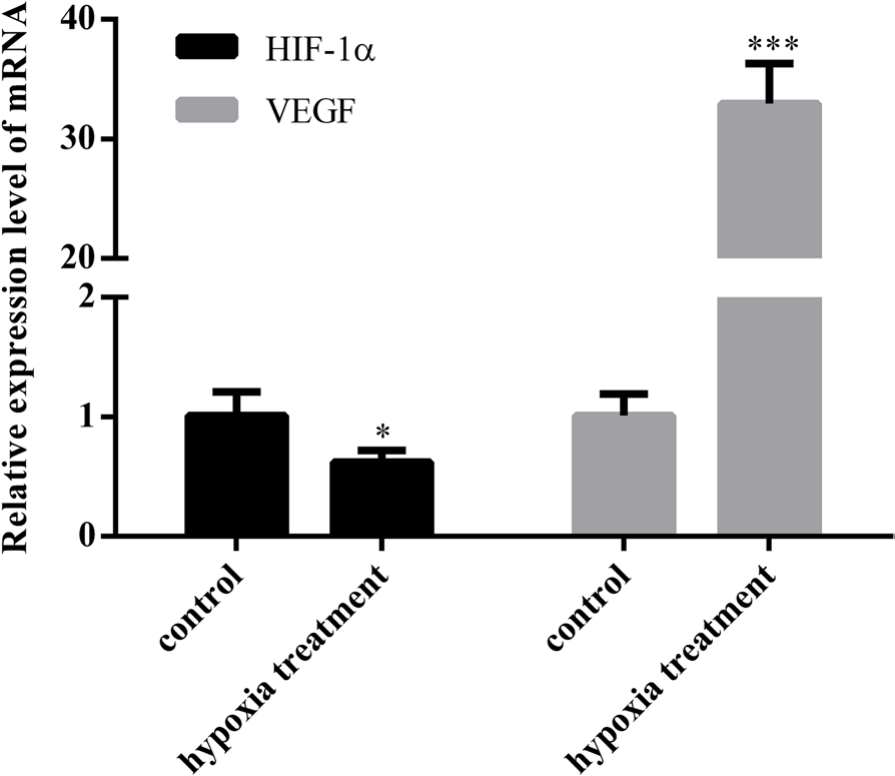
mRNA expression of HIF-1α and VEGF in HTR8 cells treated with hypoxia and normal culture (*P < 0.05, ***P < 0.001) (*n* = 3 per group). HTR8 cells were cultured under hypoxia and normal. PCR assay measured HIF-1α and VEGF expression in the cells. Data are depicted as means ± SD. Statistical significance assessed by t tests

**Fig. 6:**
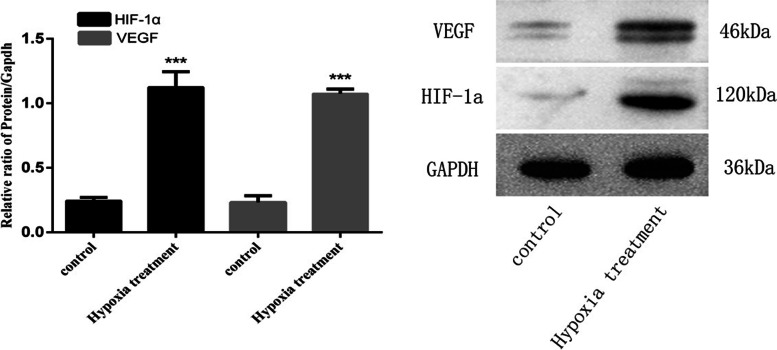
Expression of HIF-1α and VEGF protein in hypoxia treatment group and normal culture group (*P < 0.05, ***P < 0.001) (*n* = 3 per group). HTR8 cells were cultured under hypoxia and normal. Western blot assay measured HIF-1α and VEGF expression in the cells. Data are depicted as means ± SD. Statistical significance assessed by one-way ANOVA. The gels and blots displayed in the main paper are cropped

Next, we detect the expression levels of HIF-1α and VEGF in HTR8 cells after hypoxia treatment and in a normal treatment group (Fig. 5). The mRNA expression levels of HIF-1α in HTR8 cells after hypoxia treatment was significantly lower than those in the normal treatment group [control vs. PE = (1.01 ± 0.20) vs. (0.62 ± 0.10), *P* = 0.0403], while the mRNA expression levels of VEGF was significantly higher than those in the normal treatment group [control vs. PE = (1.01 ± 0.18) vs. (32.94 ± 3.36), *P* = 0.0001] (Fig. 5). In contrast to mRNA expression, the expression of HIF-1α protein in HTR8 cells after hypoxia treatment was significantly higher than those in the normal treatment group. The protein expression levels of VEGF were also higher than those in the normal treatment group (Fig. 6).

#### Detection of cell proliferation ability

**Fig. 7:**
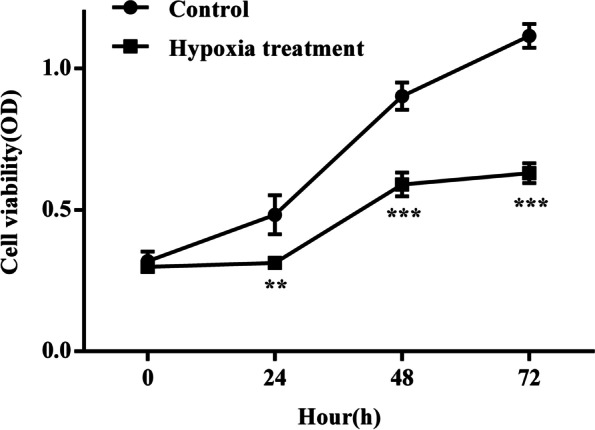
Proliferation of HTR8 cells in hypoxia treatment group and normal culture group (**P < 0.01, ***P < 0.001) (*n* = 3/group). HTR8 cells were cultured under hypoxia and normal. cell proliferation viability was tested by CCK8 assay (0 h, 24 h, 48 h, 72 h). Data are depicted as means ± SD. Statistical significance assessed by t test

To investigate whether hypoxia affected the proliferation ability of human chorionic trophoblast (HTR8) cells, we used the CCK8 assay to detect the absorbance of HTR8 cells in a hypoxia-treated group and a normal control group (at 0 h, 24 h, 48 h, 72 h). We found that the proliferation rate of HTR8 cells in the hypoxia treatment group was significantly slower than that in the control group (Fig. 7). Collectively, these results showed that hypoxia inhibited the proliferation of HTR8 cells.

#### Cell invasion ability

**Fig. 8:**
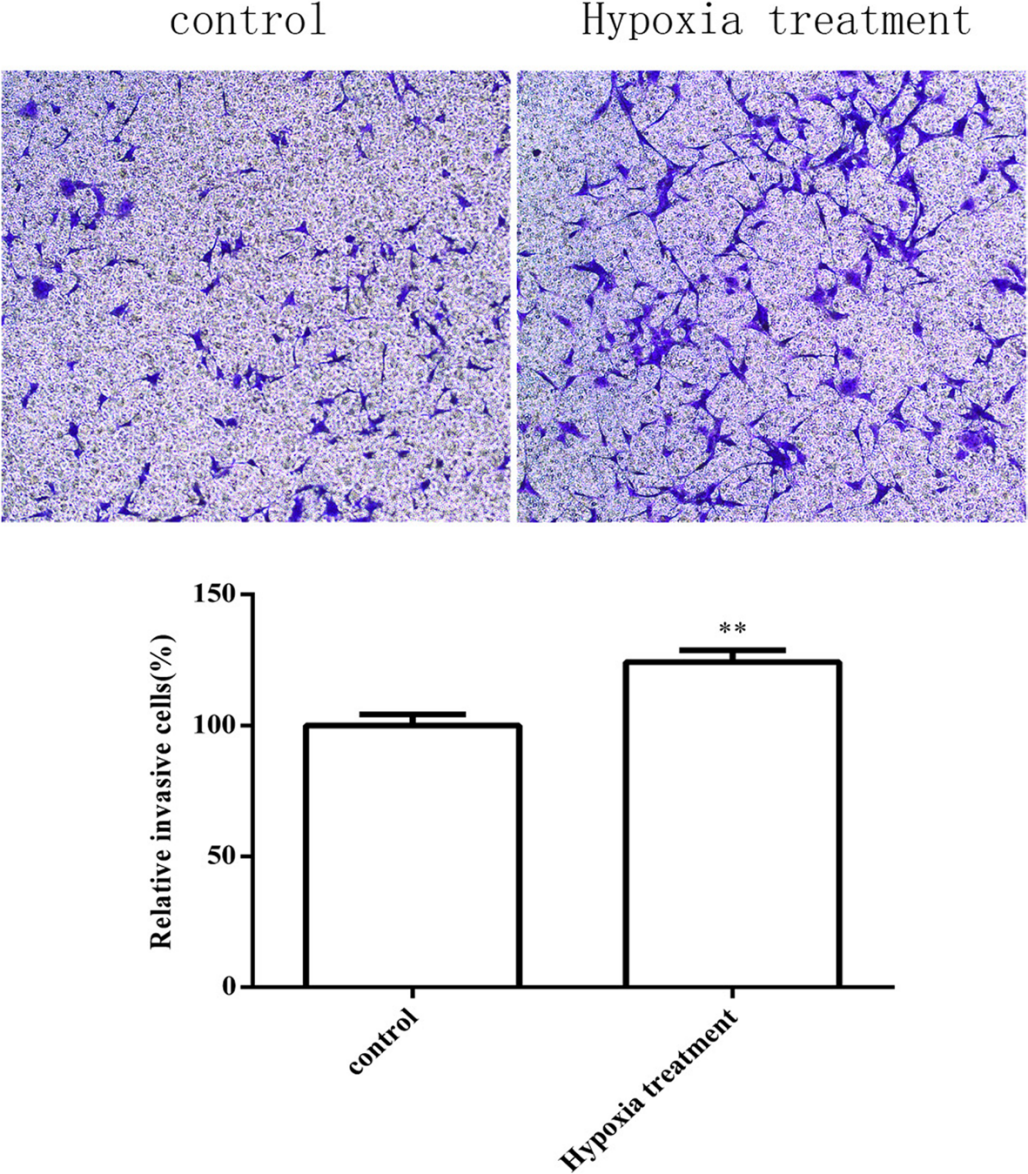
Invasion ability of HTR8 cells in hypoxia treatment group and normal culture group (100 ×) (**P < 0.01) (*n* = 3/group). HTR8 cells were cultured under hypoxia and normal. Cell invasion abilities were evaluated by transwell cell invasion assay. Data are depicted as means ± SD. Statistical significance assessed by t test

To investigate whether hypoxia affects the invasion ability of HTR8 cells, we used the Transwell method to detect the invasion ability of HTR8 cells in a hypoxia-treated experimental group and a normal control group. As shown in Fig. 8, the invasion ability of HTR8 cells in the hypoxia-treated group was significantly higher than that in the control group (*P* < 0.01). Collectively, these results showed that hypoxia enhanced the invasion ability of HTR8 cells.

#### Angiogenic capacity

**Fig. 9:**
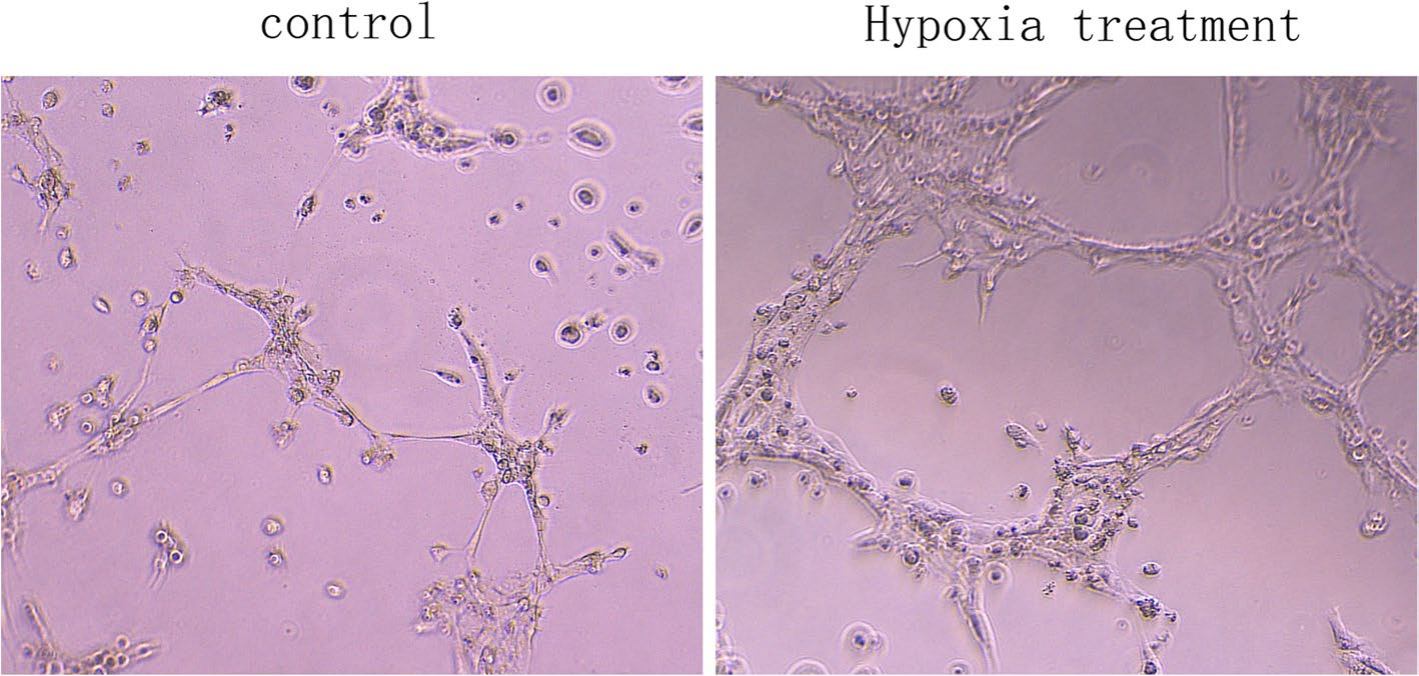
Angiogenesis of HTR8 cells in hypoxia treatment group and normal culture group (200 ×) (*n* = 2 per group). HTR8 cells were cultured under hypoxia and normal. Angiogenic capacity was detected by endovascular tube formation assay

To investigate whether hypoxia influences the vasogenesis of HTR8 cells, we used an endovascular tube formation assay to detect the number of branching HTR8 cells in the hypoxia-treated group and a normal control group. Endovascular tube formation assays showed that the number of cell branches in the experimental group was higher than that in the control group after hypoxia treatment (Fig. 9). These results showed that hypoxia enhanced the angiogenesis of HTR8 cells.

### The effects of mitochondrial ferritin (FtMt) on the proliferation and invasion of trophoblast cells after hypoxia treatment, the vascular cavity formation ability of endothelial cells, and expression of HIF-1α and VEGF

#### The infection of target cells with lentiviruses

**Fig. 10:**
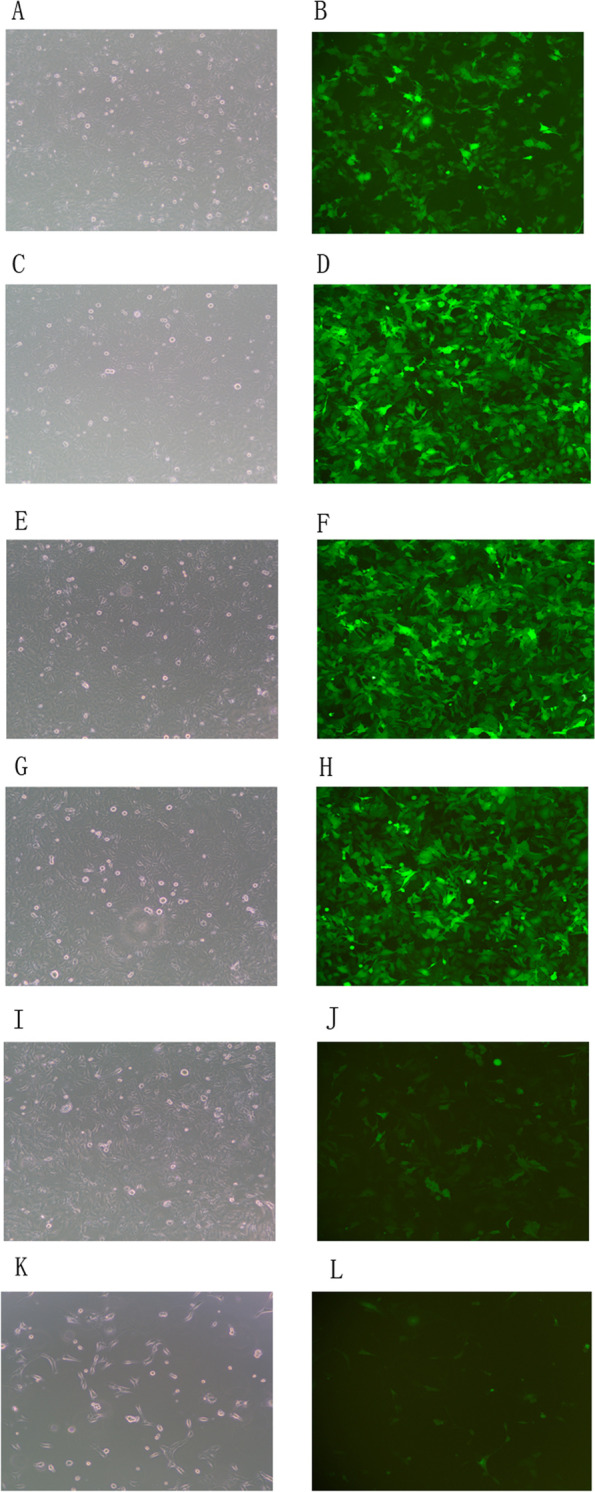
Different groups transfection efficiency. **A**, **B** the FtMt − / − negative control group; **C**, **D** the FtMt1 inhibited expression group (expressed in FtMt − / −); **E**, **F** the FtMt2 inhibited expression group (expressed in FtMt − / −); **G**, **H** the FtMt3 inhibited expression group (expressed in FtMt − / −); **I**, **J** the FtMt + / + positive control group. **K**, **L** the FtMt overexpression group (expressed in FtMt + / +); **A**, **C**, **E**, **G**, **I**, **K** the bright-field images; **B**, **D**, **F**, **H**, **J**, **L** the dark-field images

We used a lentivirus vector (293FT) and used this to generate and transfect a lentiviral FtMt1 inhibitor, FtMt2 inhibitor, FtMt3 inhibitor, FtMt-negative control (FtMt-NC-knockdown), FtMt over-expression vector, and a FtMt-positive control vector (FtMt-NC-overexpression) into a human chorionic trophoblast cell line (HTR8). The viral titer was 1 × 10^9^, and fluorescence was observed by fluorescence microscopy 72 h after transfection. The transfection efficiency was > 85% (Fig. 10).

#### HIF-1α and VEGF expression

**Fig. 11:**
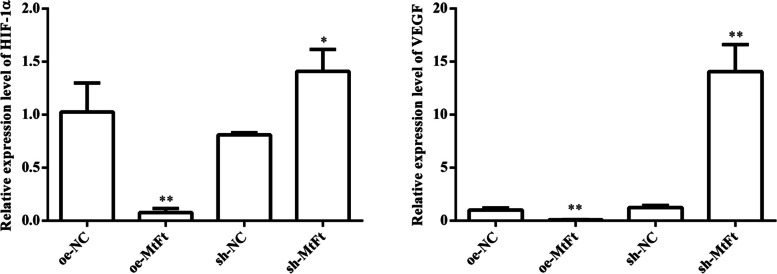
mRNA expression of HIF-1α and VEGF in HTR8 cells transfected with lentivirus oe-NC and oe-FtMt and sh-NC and sh-FtMt after hypoxia treatment (*P < 0.05, **P < 0.01) (*n* = 3 per group). HTR8 cells were cultured under hypoxia and transfected with oe-NC and oe-FtMt and sh-NC and sh-FtMt vectors. PCR assay measured HIF-1α and VEGF expression in the cells. Data are depicted as means ± SD. Statistical significance assessed by t tests. oe: overexpression, sh: inhibitor

**Fig. 12:**
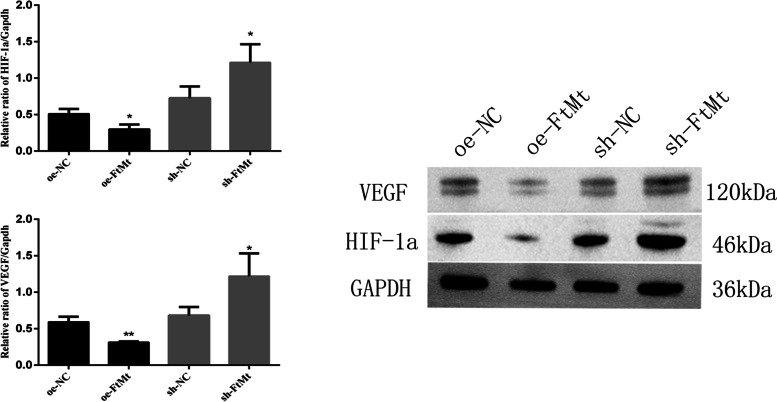
Protein expression of HIF-1α and VEGF in HTR8 cells transfected with lentivirus oe-NC and oe-FtMt and sh-NC and sh-FtMt after hypoxia treatment (*P < 0.05, **P < 0.01) (*n* = 3 per group). HTR8 cells were cultured under hypoxia and transfected with oe-NC and oe-FtMt and sh-NC and sh-FtMt vectors. Western blot assay measured HIF-1α and VEGF expression in the cells. Data are depicted as means ± SD. Statistical significance assessed by one-way ANOVA. The gels and blots displayed in the main paper are cropped

We detect the expression levels of HIF-1α and VEGF mRNA in HTR8 cells transfected with lentivirus oe-NC, oe-FtMt and sh-NC, sh- FtMt after hypoxia treatment. Ad shown in Fig. 11, the mRNA expression levels of HIF-1α and VEGF in HTR8-oe-FtMt cells were significantly lower than those in HTR8-oe-NC cells (*P* < 0.01). Furthermore, the mRNA expression levels of HIF-1α and VEGF in HTR8- sh-FtMt cells were significantly higher than those in HTR8-sh-NC cells (*P* < 0.05). And the protein expression levels of HIF-1α and VEGF in HTR8-oe-FtMt cells were significantly lower than those in HTR8-oe-NC cells (*P* < 0.05). Furthermore, the protein expression levels of HIF-1α and VEGF in HTR8-sh-FtMt cells were significantly higher than those in HTR8-sh-NC cells (*P* < 0.01) (Fig. 12).

#### Detection of cell proliferation ability

**Fig. 13:**
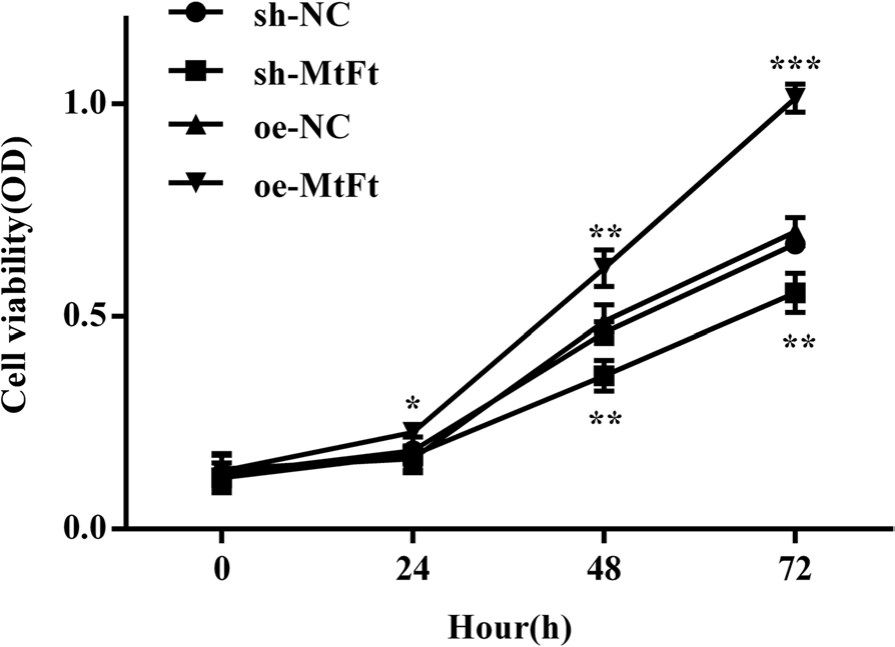
Proliferation of HTR8 cells treated with hypoxia and were transfected with lentivirus oe-NC, oe-FtMt and sh-NC, sh-FtMt (*P < 0.05, **P < 0.01, ***P < 0.001) (*n* = 3 per group). HTR8 cells were cultured under hypoxia and transfected with oe-NC and oe-FtMt and sh-NC and sh-FtMt vectors. cell proliferation viability was tested by CCK8 assay (0 h, 24 h, 48 h, 72 h). Data are depicted as means ± SD. Statistical significance assessed by t test

To investigate whether FtMt affected the proliferation of HTR8 cells, we used CCK8 assays to detect the absorbance (0 h, 24 h, 48 h, 72 h) of HTR8 cells transfected with lentivirus oe-NC, oe-FtMt and sh-NC, sh-FtMt after hypoxia treatment. We found that proliferation rate of cells in the HTR8-oe-FtMt group was significantly faster than that in the control group while the proliferation in the HTR8-sh-FtMt group was significantly slower than that in the control group (Fig. 13). These results showed that a reduction in FtMt expression inhibited the proliferation of HTR8 cells after hypoxia treatment while the overexpression of FtMt enhanced the proliferation of HTR8 cells.

#### Cell invasion ability

**Fig. 14:**
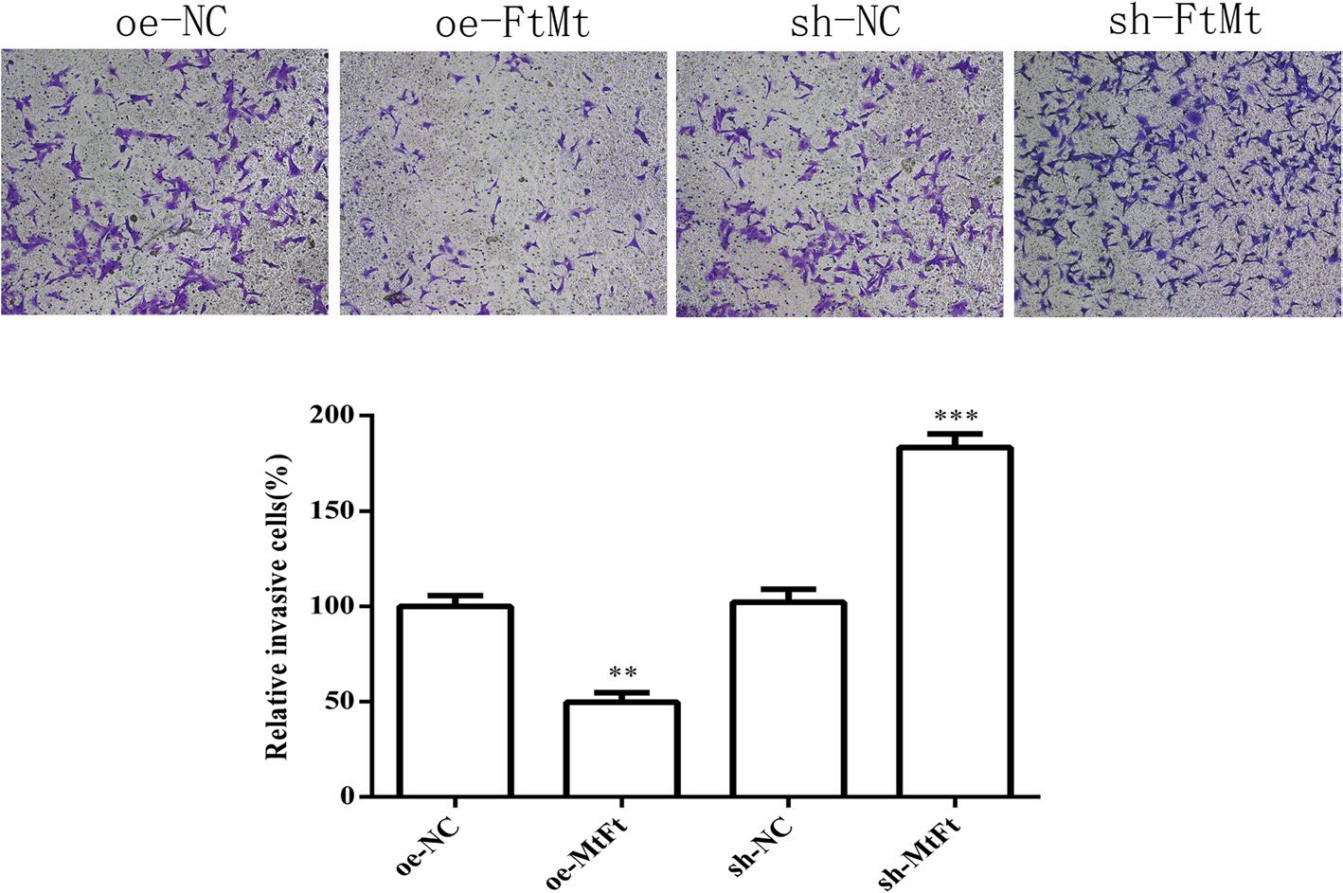
Invasion ability of HTR8 cells treated with hypoxia and were transfected with lentivirus oe-NC, oe-FtMt and sh-NC, sh-FtM (100 ×) (**P < 0.01, ***P < 0.001) (*n* = 3 per group). HTR8 cells were cultured under hypoxia and transfected with oe-NC and oe-FtMt and sh-NC and sh-FtMt vectors. Cell invasion abilities were evaluated by transwell cell invasion assay. Data are depicted as means ± SD. Statistical significance assessed by t test

To investigate whether FtMt affects the invasion ability of HTR8 cells, we used Transwell assays to detect the invasion ability of HTR8 cells transfected with lentivirus oe-NC, oe-FtMt and sh-NC, sh-FtMt after hypoxia treatment. As shown in Fig. 14, the invasion ability of the HTR8-oe-FtMt group was significantly lower than that of the control group while the invasion ability of the HTR8-sh-FtMt group was significantly higher than that of the control group (*P* < 0.01). These results showed that a reduction in the expression of FtMt enhanced the invasion ability of HTR8 cells after hypoxia treatment while the overexpression of FtMt inhibited the invasion ability of HTR8 cells.

#### Angiogenic capacity

**Fig. 15:**
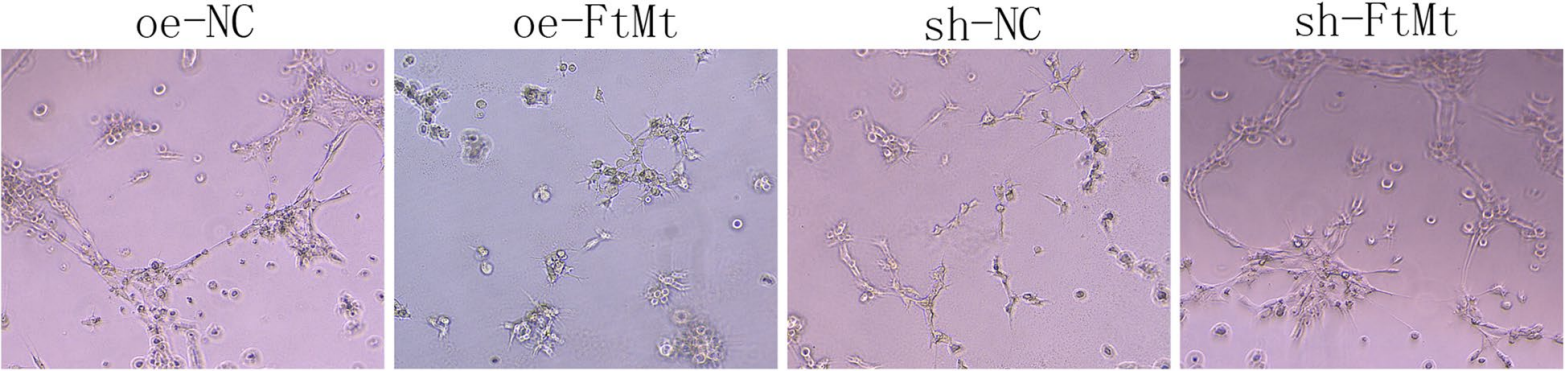
Angiogenesis of HTR8 cells treated with hypoxia and were transfected with lentivirus oe-NC, oe-FtMt and sh-NC, sh-FtMt (200 ×) (*n* = 2 per group). HTR8 cells were cultured under hypoxia and transfected with oe-NC and oe-FtMt and sh-NC and sh-FtMt vectors. Angiogenic capacity was detected by endovascular tube formation assay

To investigate whether FtMt affected vasogenesis in HTR8 cells, we used endovascular tube formation assays to detect the number of branching cells in each group of HTR8 cells transfected with lentivirus oe-NC, oe-FtMt and sh-NC, sh-FtMt after hypoxia treatment. Endovascular tube formation assays showed that the number of cell branches in the HTR8-oe-FtMt group was significantly lower than that in the control group while the number of cell branches in the HTR8- sh-FtMt group was significantly higher than that in the control group (Fig. 15). These results showed that a reduction in the expression of FtMt inhibited the angiogenesis of HTR8 cells after hypoxia treatment while the overexpression of FtMt enhanced angiogenesis in HTR8 cells.

## Discussion

Preeclampsia (PE) is a multifactorial vascular disease caused by oxidative stress injury, inflammatory response, and angiogenesis imbalance [21, 22]. Some studies have suggested that one of the key pathological changes of PE is placental hypoxia [23, 24]. These results indicate that oxygen saturation is critical for supporting the placenta and normal pregnancy. We suggest that superficial trophoblast invasion and spiral artery remodeling lead to continuous placental hypoxia which upregulates HIF-1α expression, thus leading to endothelial dysfunction and the pathogenesis of PE [25]. In recent years, an increasing number of studies have suggested that changes in placental vascular development are closely related to PE and that vascular endothelial growth factor (VEGF) plays a key role in angiogenesis and participates in placental development [10, 11, 26]. As one of the most important downstream target genes of HIF-1α, VEGF is a vascular permeability factor that is particularly important in endothelial cells [12]. VEGF and HIF-1α are both important factors involved in the pathophysiology of PE; research has also shown that the HIF-1α/VEGF axis plays an important role in regulating and maintaining the integrity of the normal placental barrier [27].

In this study, we compared HIF-1α and VEGF expression levels in placental tissues from PE and normal pregnancies. Similar to previous studies [28], PCR results showed that the expression levels of HIF-1α and VEGF in clinical placental tissue samples in the PE group were significantly higher than those in the normal control group. Although some studies have shown that the expression levels of VEGF in the placenta of patients with PE are lower than those in normal pregnancy placenta tissues [29–31], some existing studies reported findings that are consistent with our present results. The difference between early-onset PE and late-onset PE may be one of the reasons that influence the above results. Studies have shown that patients with PE exhibit elevated levels of serum VEGF expression and an imbalance in angiogenesis [32–35]. Conversely, other studies have reported reduced expression levels of VEGF throughout the body [36–38]. These seemingly contradictory results may be due to the different research methods adopted by different authors. And the controversial results of VEGF expression in PE placenta may also be due to the varying severity of PE included in different studies. In addition, we compared the gestation weeks of cases in the study population, and found that the PE group was significantly lower than the control group. This may also have an impact on HIF-1α and VEGF expression levels, as the placental expression of many genes/proteins can vary during gestation.

PE is a pregnancy complication that is characterized by superficial trophoblast infiltration and placental abnormalities, thus resulting in persistent hypoxia of the placenta and the release of various mediators into the maternal circulation. [39] In this study, human chorionic trophoblast cells (HTR8) were treated with hypoxia in a cell culture model to simulate the onset of PE; we then investigated the effects of hypoxia on HTR8 cell proliferation, apoptosis and angiogenesis. Considering that the pathogenesis of PE is mainly trophoblast cell lesions, we used HTR8 cells in this study. The results of qRT-PCR showed that the mRNA expression levels of HIF-1α in HTR8 cells after hypoxia treatment were significantly lower than those in the normal treatment group while the mRNA expression levels of VEGF were significantly higher than those in the normal treatment group. The results of western blotting showed that, in contrast to the mRNA expression results, the protein expression levels of HIF-1α and VEGF in HTR8 cells after hypoxia treatment were significantly higher than those in the normal treatment group; these results were consistent with previous studies of expression levels in placental tissues from PE patients. We speculated that this might be due to the inconsistent transcription and translation levels of HIF-1α in HTR8/SVneo cells under hypoxia induction. In fact, the relationship between gene transcription and translation is complicated. Firstly, the time and locus of transcription and translation of gene expression exist spatio-temporal intervals. Secondly, after transcription, there will be post-transcription processing, degradation of transcription products, translation, post-translation processing and modification of several levels. Therefore, transcription level and translation level are not completely consistent; Again, due to the different time points of detection, the mRNA may have been degraded when the protein reached the peak or the protein amount was still increasing when the mRNA reached the peak, thus the protein level was inconsistent with the mRNA level [26, 40]. CCK8 assays showed that the proliferation rate of HTR8 cells in the hypoxia treatment group were significantly slower than that in the control group, thus indicating that hypoxia could inhibit the proliferation of HTR8 cells. Similarly, Transwell assays results showed significantly higher levels in hypoxia treatment, not in controls, thus indicating that hypoxia could enhance the invasion ability of HTR8 cells. Finally, the results of endovascular tube formation assays showed that the number of branching HTR8 cells in the hypoxia treatment group was significantly higher than that in the control group, thus suggesting that hypoxia could enhance angiogenesis in HTR8 cells. Our results suggest that hypoxia-treated HTR8 cells mimic the onset of PE, and that this treatment significantly altered the expression levels of HIF-1α and VEGF while exerting certain effects on cell proliferation, apoptosis and angiogenesis, thus highlighting the potential role of hypoxia-induced HIF-1α and VEGF in the development of PE.

Mitochondria are multi-functional organelles that are found in eukaryotic cells and play key roles in ATP production, calcium homeostasis, free radical production, cell proliferation, apoptosis, and necrosis [41]. Placental mitochondria are located in the gas transport region and are responsible for providing ATP to the placental tissues that mediate fetal growth, nutrient transport, and ion regulation [42]. In a previous study, Myatt [43] suggested that placental mitochondrial dysfunction in PE may be caused by significant changes in mitochondrial function caused by long-term hypoxia or continuous ischemia/reoxygenation, thus resulting in the inability of cells to maintain ATP production, excessive ROS production, and mitochondrial damage. Since the late 1980s, it has been suggested that PE might represent a form of mitochondrial disease [44–46].

Mitochondrial ferritin (FtMt) is a newly discovered H-ferritin-like protein that is only expressed in the mitochondria. A recent study found that FtMt expression is related to reduced mitochondrial metabolic activity and glutathione level, as well as the accompanying reactive oxygen species level and increased apoptosis [47]. Other studies have shown that FtMt reduces oxidative stress damage in various cell models by reducing the excessive production of toxic iron [48, 49]. Another study found that FtMt can protect cells from oxidative stress by regulating the production of unstable iron and ROS in the mitochondria [50].

This was the first study to investigate the expression levels of FtMt in placental tissues from PE and normal pregnancies and found that FtMt was highly expressed in the placental tissues of patients with PE. Similar to the study reported by Wang [15] et al., the upregulation of FtMt level in ischemic brain of mice also confirmed that the upregulation of FtMt expression can protects cells in tissues from iron-dependent oxidative damage. Then, we constructed FtMt inhibitor or FtMt over-expression lentiviral vectors and used these to infect HTR8 cells. This allowed us to investigate the effect of FtMt on cell function after hypoxia treatment, thus reflecting its role in the pathogenesis of preeclampsia. Fluorescence quantitative PCR further showed that the mRNA expression levels of HIF-1α and VEGF in HTR8-oe-FtMt cells were significantly lower than those in HTR8-oe-NC cells. The mRNA expression levels of HIF-1α and VEGF in HTR8-sh-FtMt cells were significantly higher than those in HTR8-sh-NC cells. The protein expression levels of HIF-1α and VEGF in HTR8-oe-FtMt cells were significantly lower than those in HTR8-oe-NC cells. The protein expression levels of HIF-1α and VEGF in HTR8-sh-FtMt cells were significantly higher than those in HTR8-sh-NC cells. Studies have suggested that FtMt overexpression can reduce the expression levels of HIF-1α and VEGF in hypoxia treated cells, and conversely, inhibition of FtMt expression can increase the expression levels of HIF-1α and VEGF in hypoxia treated cells, thus suggesting that FtMt may play a critical role in the development of PE by influencing the expression of HIF-1α/VEGF. CCK8 assays further showed that the reduced expression of FtMt inhibited the proliferation of HTR8 cells after hypoxia treatment while the overexpression of FtMt enhanced the proliferation of HTR8 cells. Transwell assays further showed that the reduced expression of FtMt enhanced the invasion ability of HTR8 cells after hypoxia treatment while the overexpression of FtMt inhibited the cell invasion ability of HTR8 cells. The results of endovascular tube formation assays further showed that the reduced expression of FtMt inhibited the angiogenesis of HTR8 cells after hypoxia treatment while the overexpression of FtMt enhanced angiogenesis in HTR8 cells. These results suggest that the expression of FtMt can affect the proliferation, invasion and angiogenesis of HTR8 cells after hypoxia treatment and may play a role in the process of mitochondrial oxidative stress in PE. A previous study also reported that FtMt plays a role in regulating iron and oxidative stress responses in cells [51].

Another study reported that FtMt can inhibit mitochondrial damage by maintaining mitochondrial membrane potential and protecting the integrity of the mitochondrial membrane. FtMt can also inhibit increases in unstable iron, reduce the production of reactive oxygen species, and reduce apoptosis [52]. This suggests that FtMt plays an important role in the specific diseases by inhibiting iron accumulation and oxidative stress in cells. Similarly, Fuhrmann [53] et al. indicated that under hypoxia FtMt is protective, which can interfere with ferroptosis and improve cell viability. Collectively, these data proved that FtMt plays an important protective role in oxidative stress, which is a potential mechanism of PE.

In summary, our data suggest that FtMt expression significantly alters the expression levels of HIF-1α and VEGF and has a significant effect on the functional activity of human chorionic trophoblast cells under hypoxia. This effect is most likely mediated by the HIF-1α/VEGF axis. Ex vivo studies have demonstrated changes of FtMt expression in placental tissues from PE and normal pregnancies. Therefore, we hypothesize that FtMt may play a role in the pathogenesis of PE by acting on the HIF-1α/VEGF pathway to protect mitochondria from oxidative stress damage. However, there are still limitations in this study that need to be considered. First, the sample size of the case–control study is still small, and we should expand the sample size in future studies to better prove this research. And then, HTR8 is not pure trophoblast and the need for furthering the work by using primary trophoblast or placental organoids are required. In addition, we did not carry out ex vivo experiments in a mouse model to verify our findings and fully explain the direct role of FtMt in the pathogenesis of PE. Third, gestational week of delivery may be a limitation of this study. Last, there were no further evaluation on the correlation between this mechanism and early-onset preeclampsia and late-onset preeclampsia and the severity of PE. Therefore, further research on FtMt is now needed to enrich our understanding of the pathogenesis of PE. Our current research indicates that FtMt represents a new target for the treatment of PE.

## Conclusion

Analyses showed that FtMt plays a key role in the vascular regulation of PE trophoblast cells after hypoxia possibly acting via the HIF-1α/VEGF signaling pathway. These results provide us an enhanced understanding of the pathogenesis of PE and suggest that the HIF-1α/VEGF signaling pathway represents a new target for the treatment of PE.

## Supplementary Information

**Additional file 1.** Supplementary file.

## Data Availability

The datasets used and analysed during the current study are available from the corresponding author on reasonable request.
