# Supplementary material for: FtMt reduces oxidative stress-induced trophoblast cell dysfunction via the HIF-1α/VEGF signaling pathway
Source: BMC Pregnancy Childbirth. 2023 Mar 1;23:131. doi: 10.1186/s12884-023-05448-1 (PMC9976428; doi:10.1186/s12884-023-05448-1)

Supplementary Table Detail characteristics of all PE cases

case	Highest SBP (mmHg)	Highest DBP (mmHg)	24h urine protein	Early onset or late onset PE	Mild or severe PE
1	155	100	467	late onset	mild
2	160	108	1235	late onset	severe
3	148	98	348	late onset	mild
4	164	113	3567	early onset	severe
5	160	115	589	late onset	severe
6	166	106	943	late onset	severe
7	152	102	345	late onset	mild
8	155	105	668	late onset	mild
9	168	116	4455	early onset	severe
10	149	107	396	late onset	mild

DBP, Diastolic blood pressure.

SBP, Systolic blood pressure.

PE, preeclampsia.

Fig 6

GAPDH-1

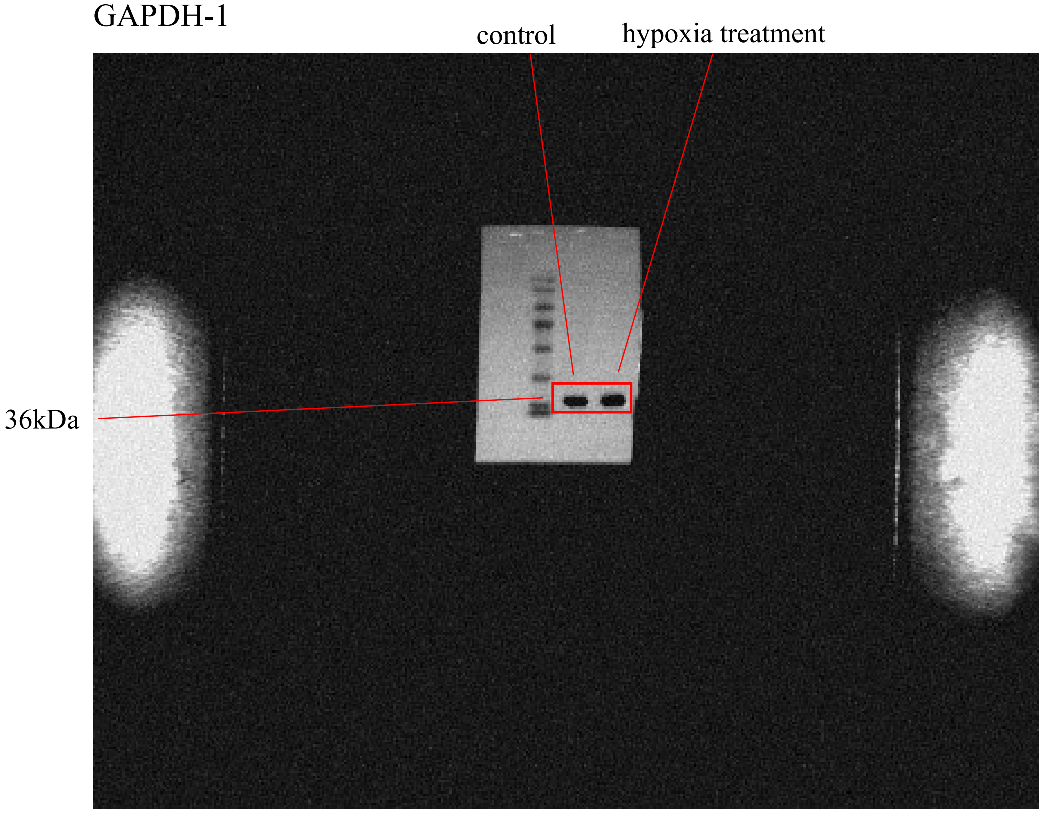

GAPDH-2

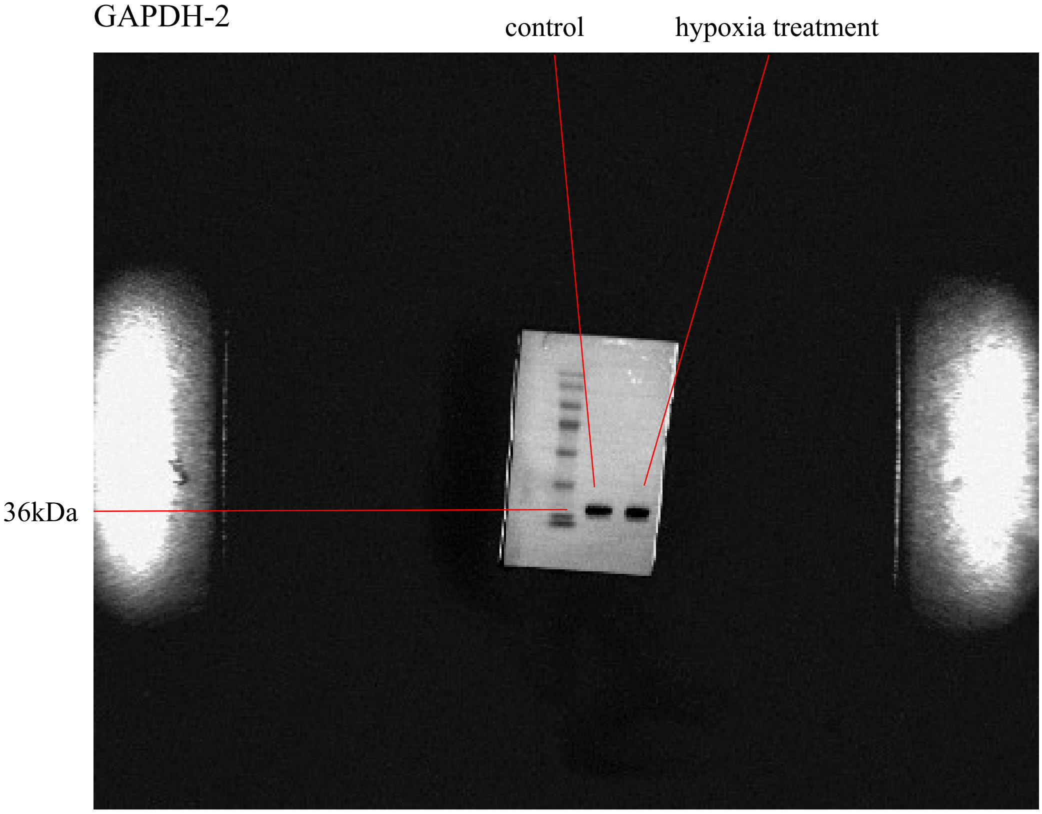

GAPDH-3

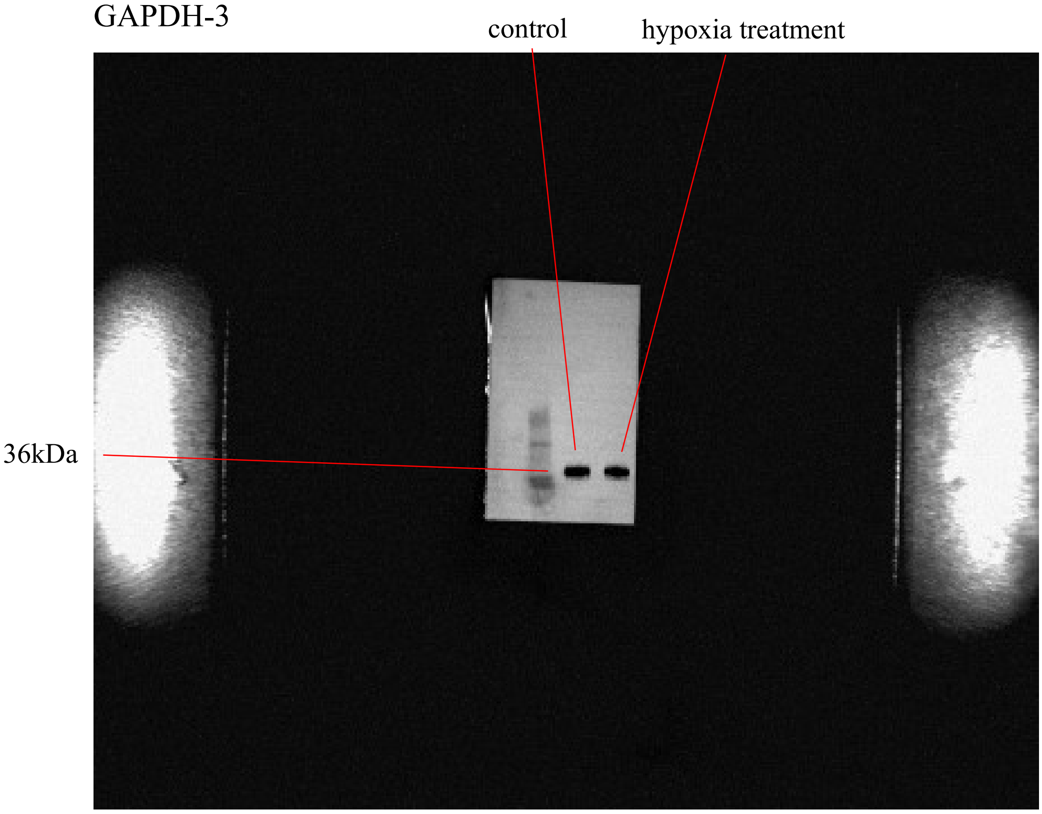

HIF-1a-1

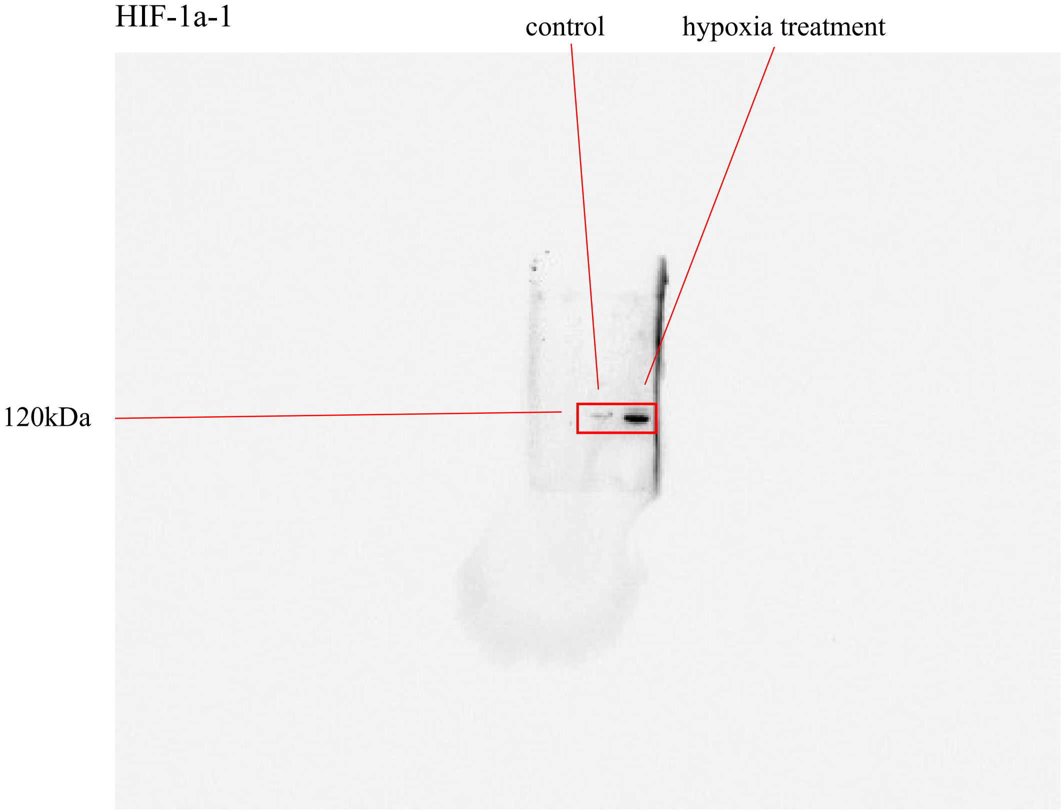

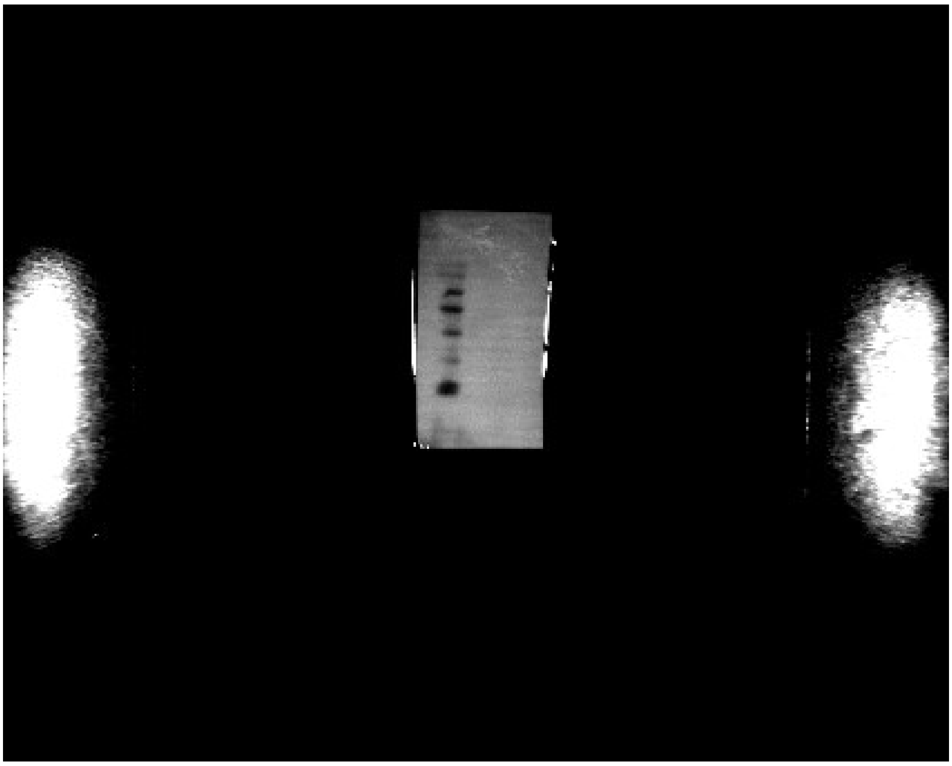

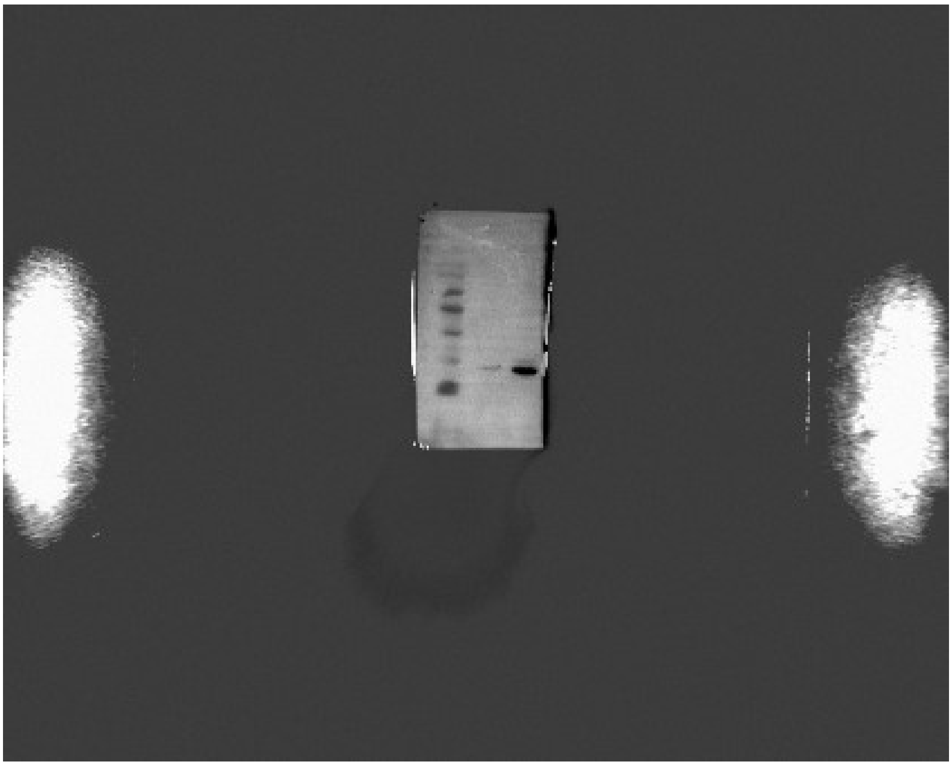

HIF-1a-2

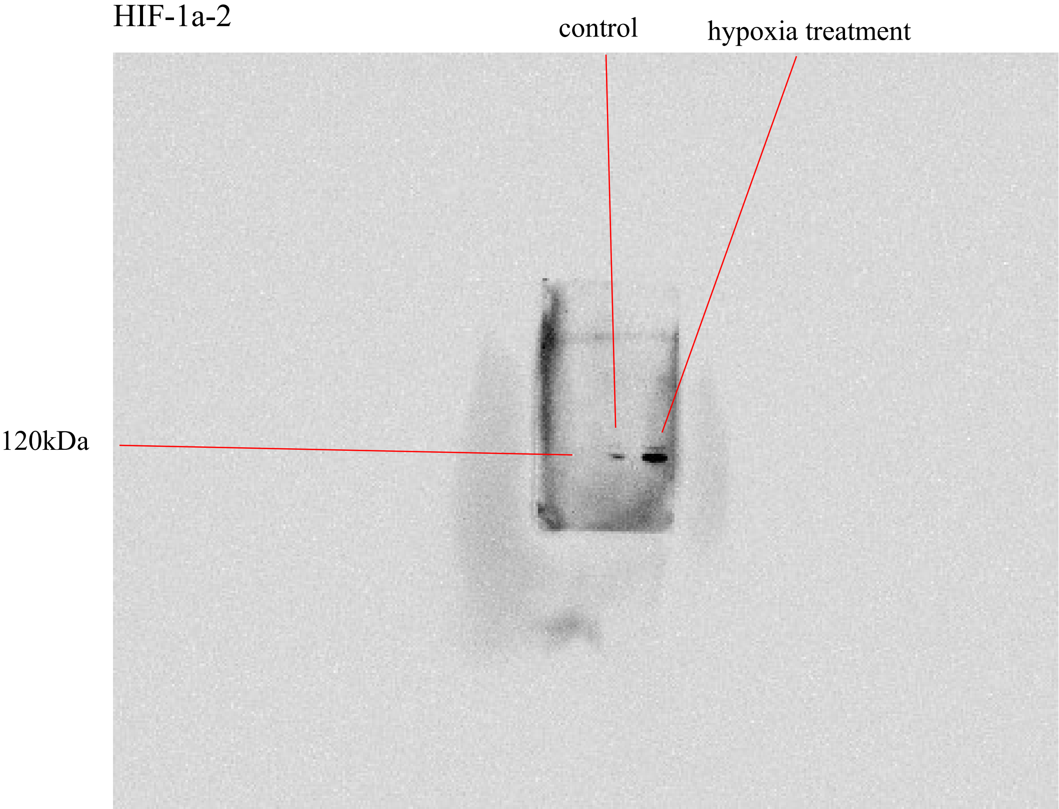

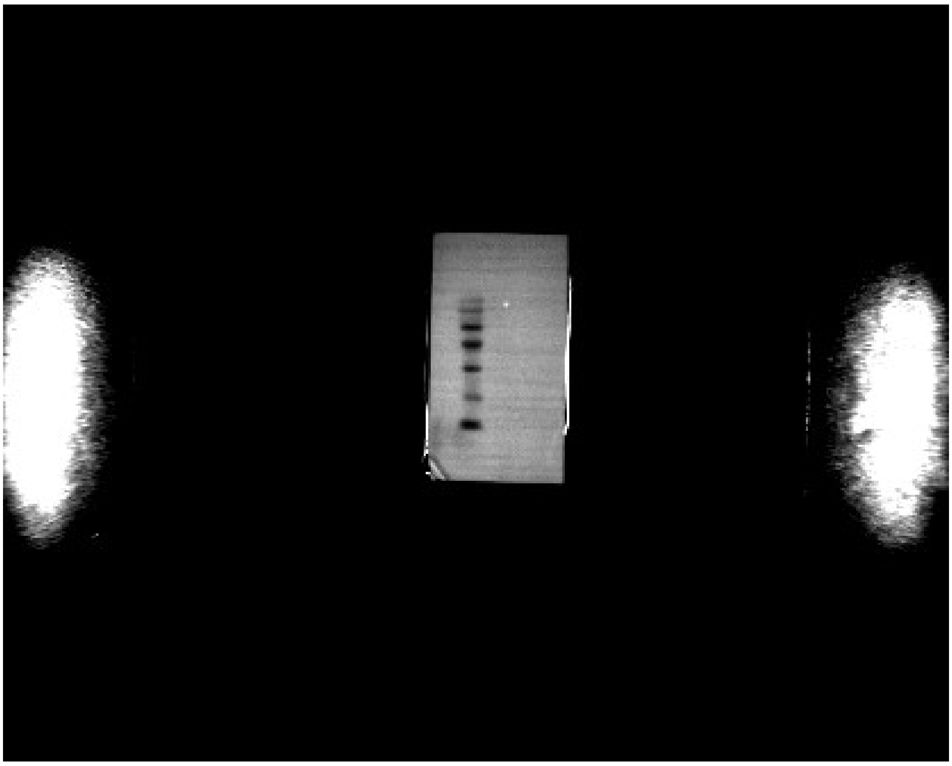
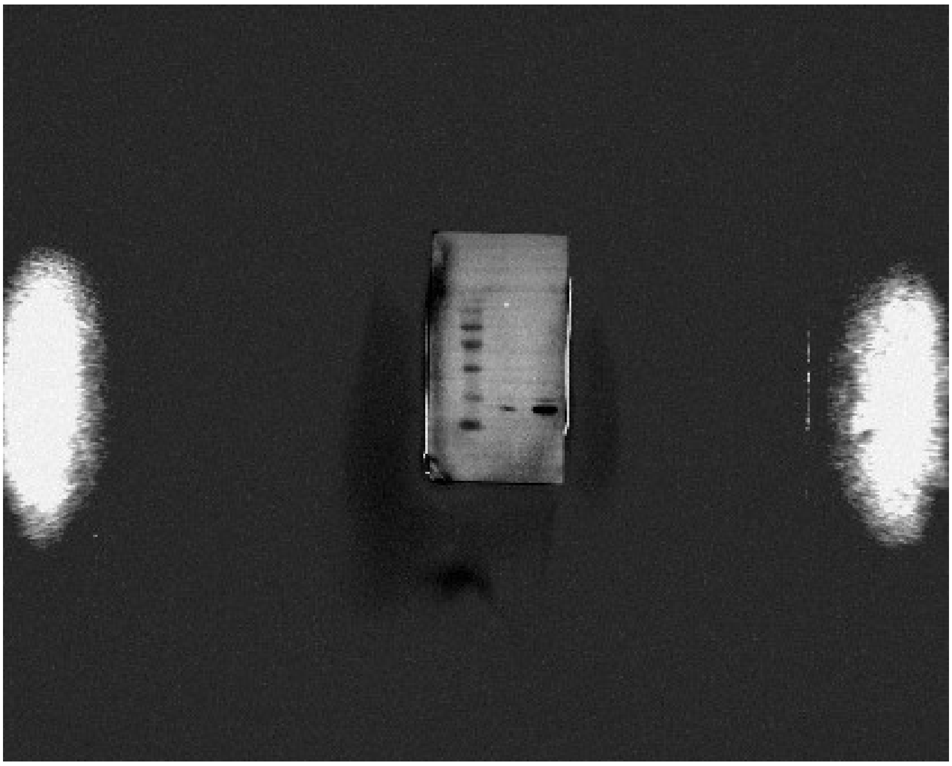

HIF-1a-3

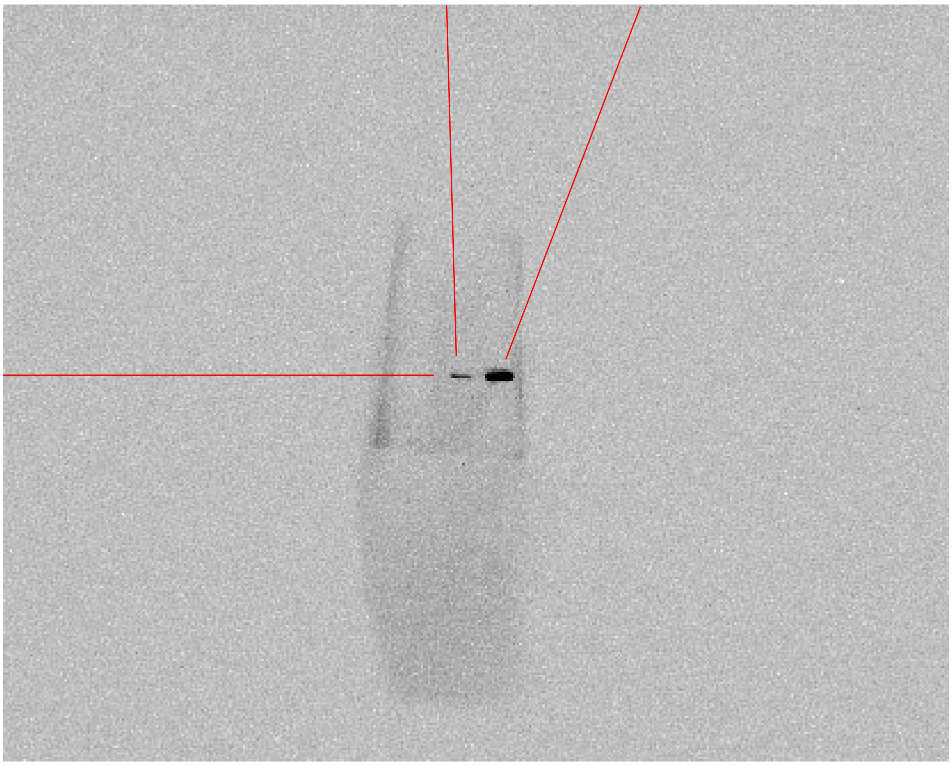

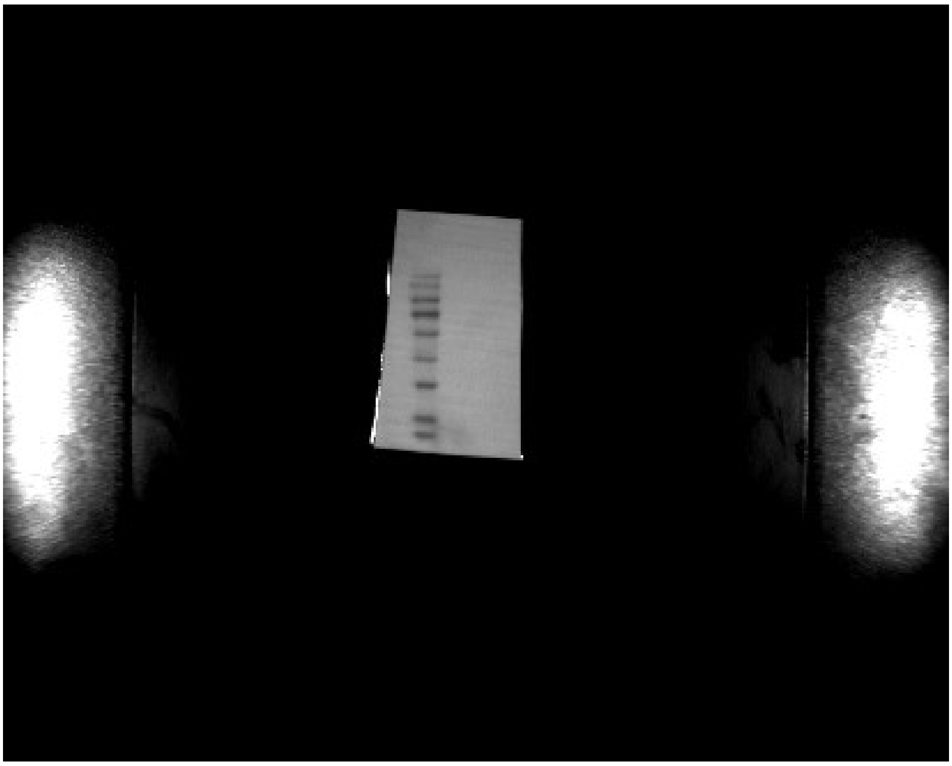

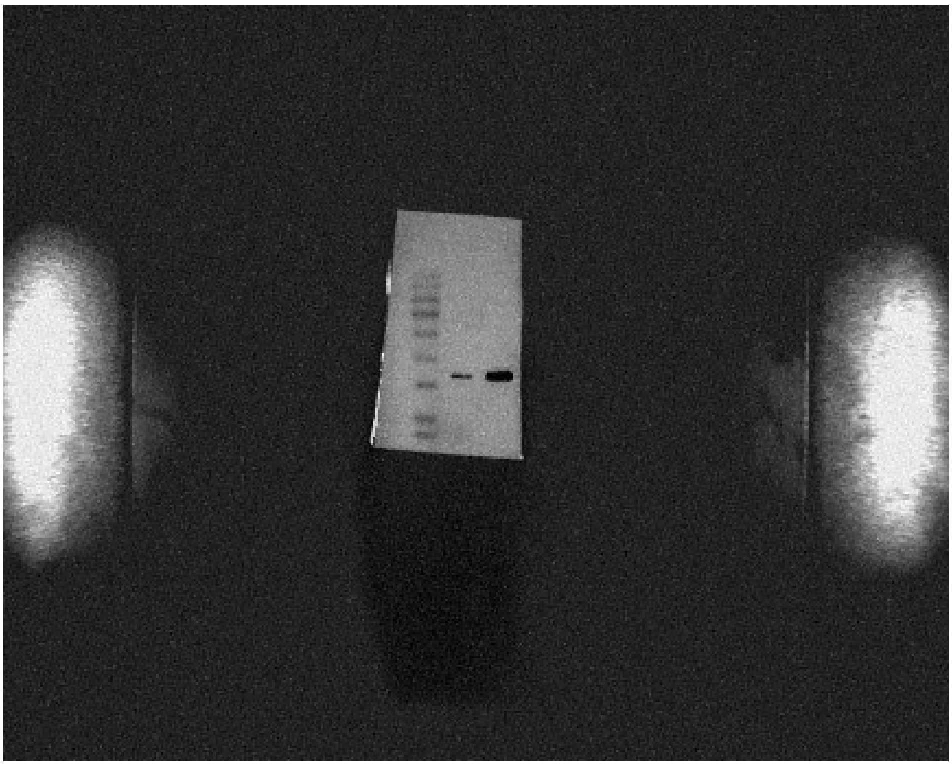

VEGF-1

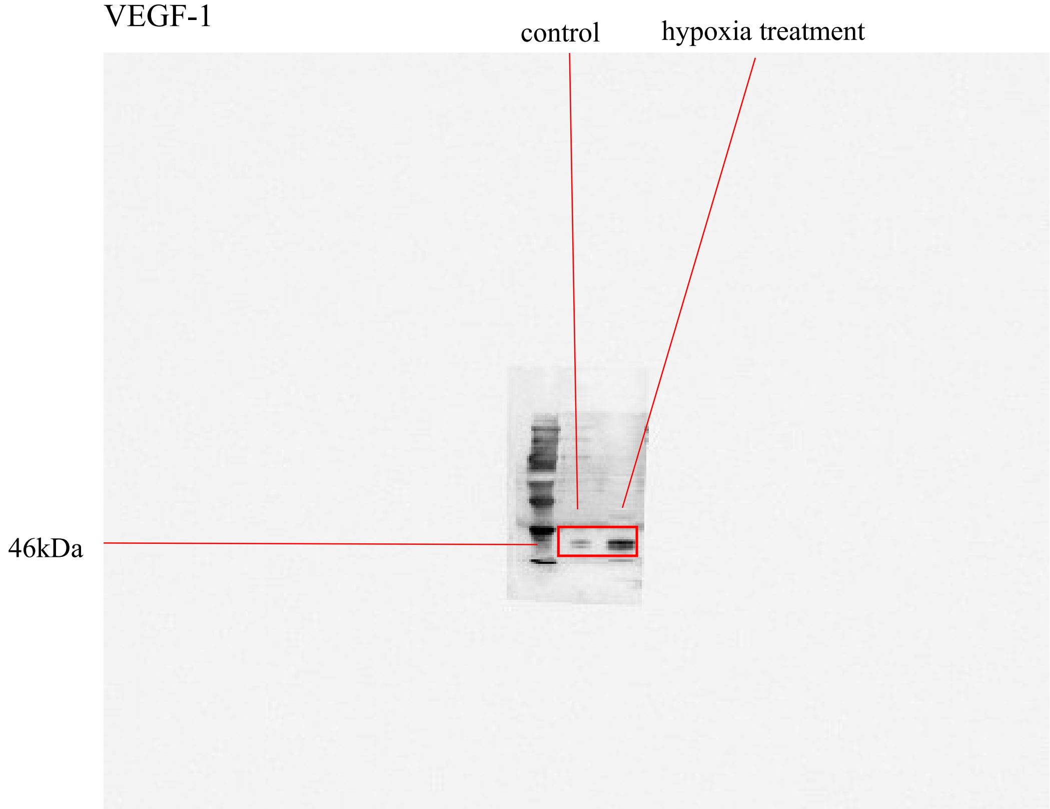

VEGF-2

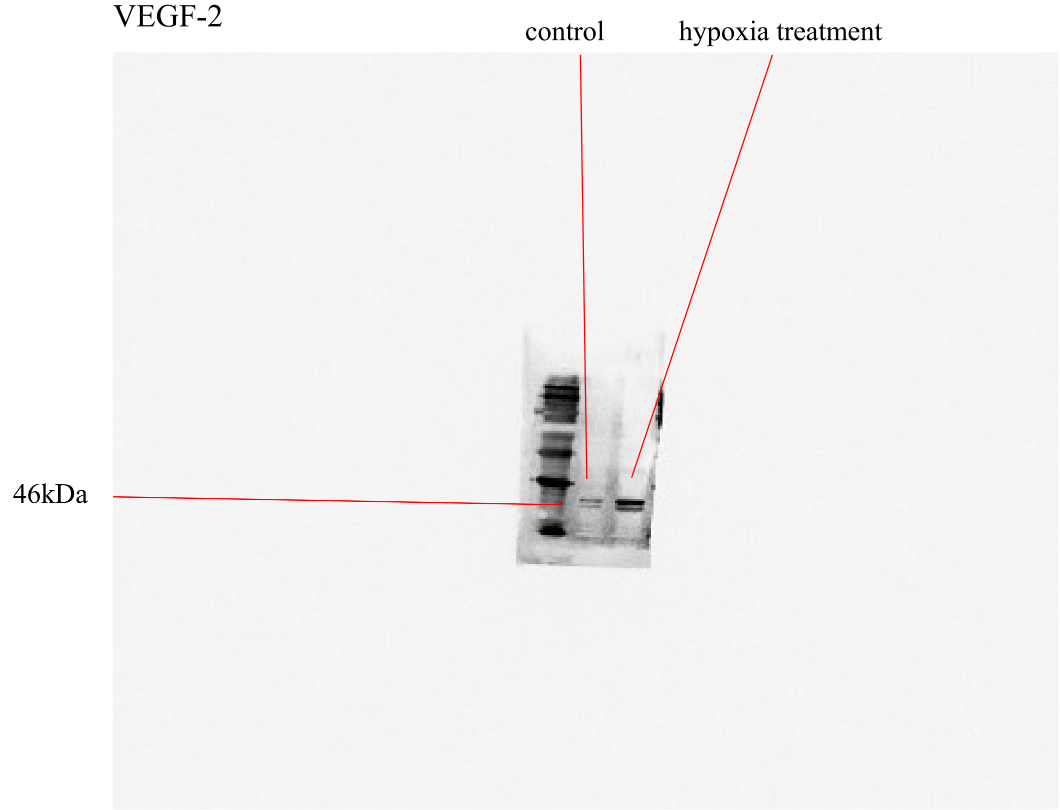

control
hypoxia treatment
46kDa

VEGF-3

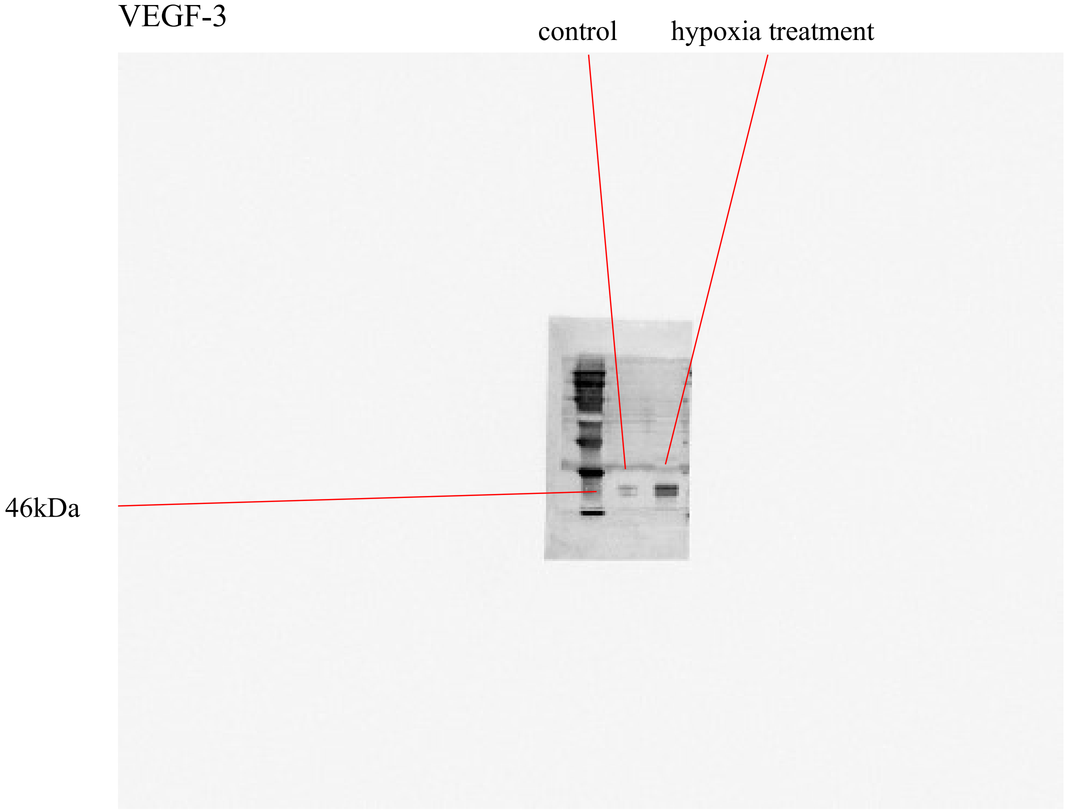

control
hypoxia treatment
46kDa

Fig 12

GAPDH-1

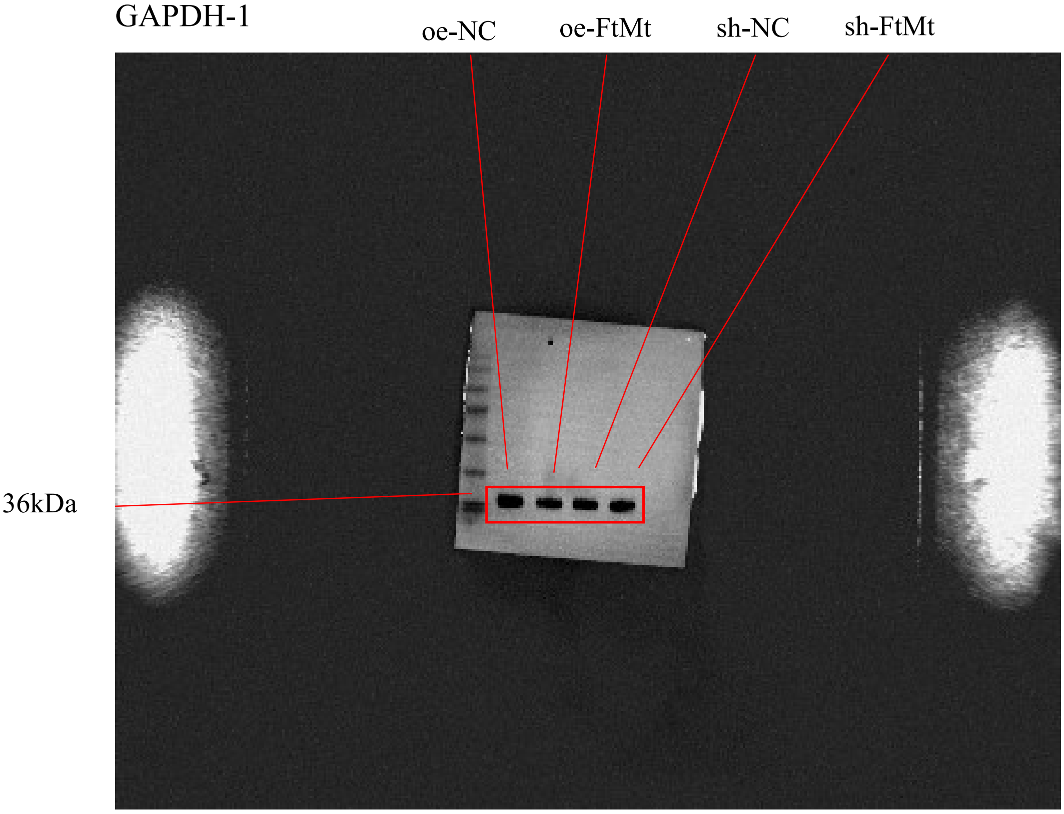

GAPDH-2

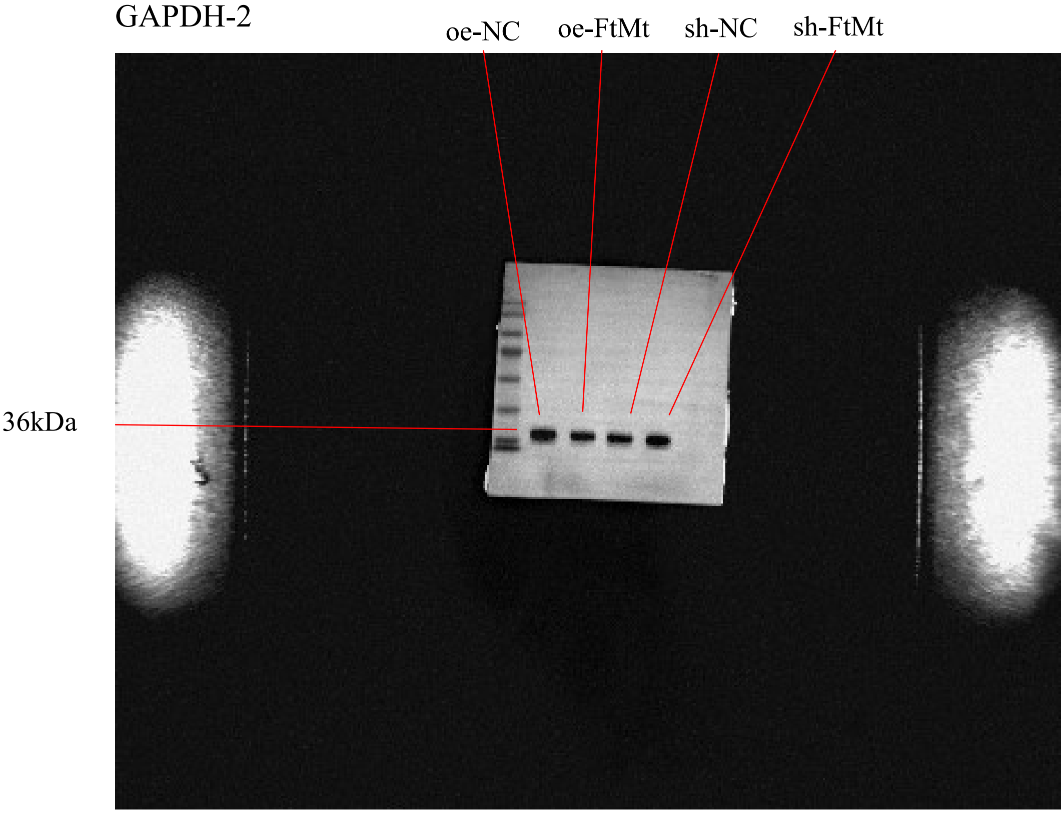

GAPDH-3

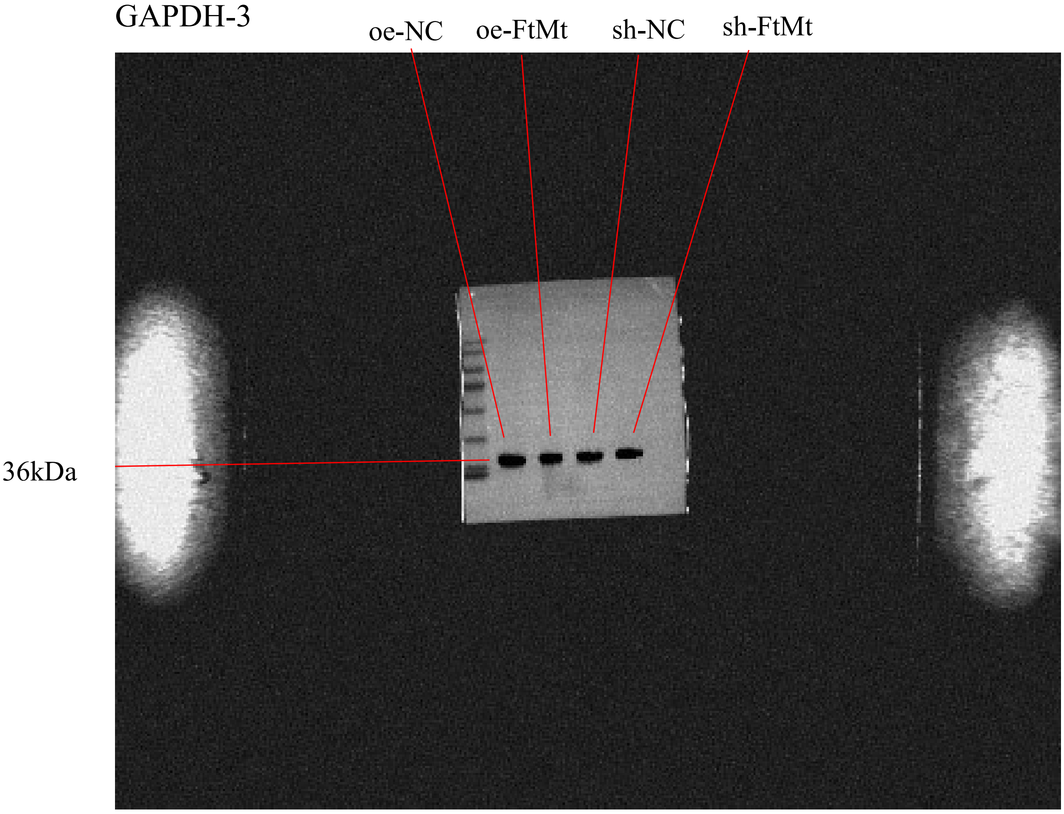

HIF-1a-1

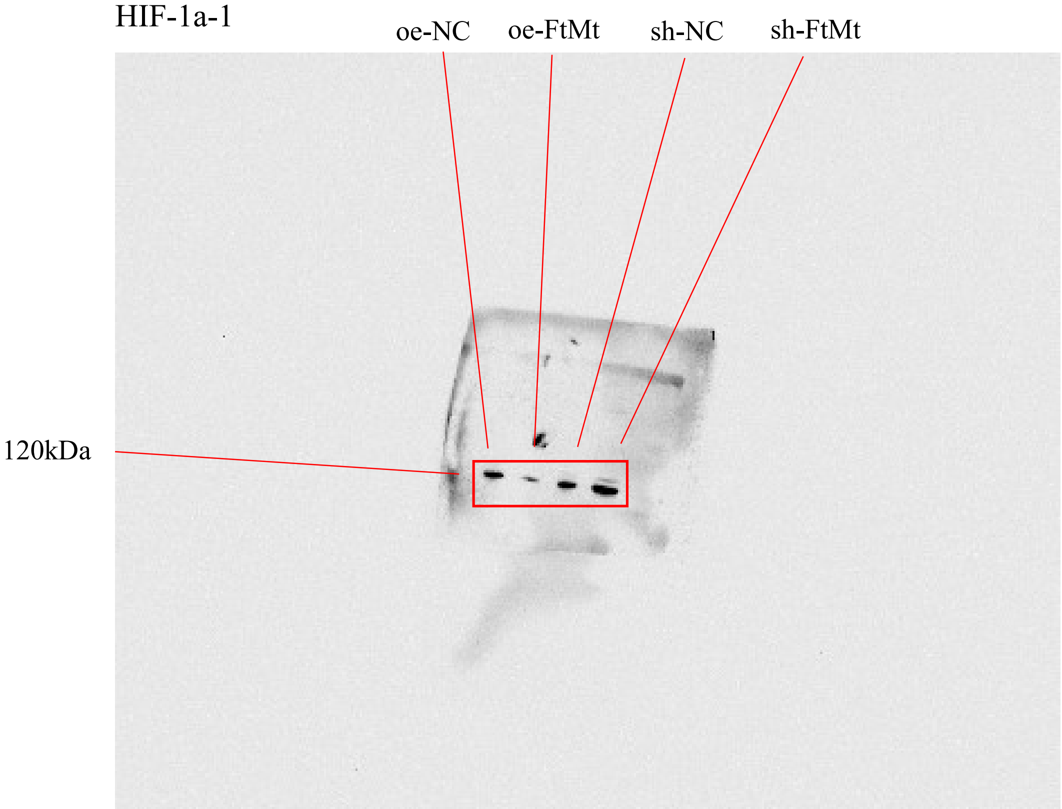

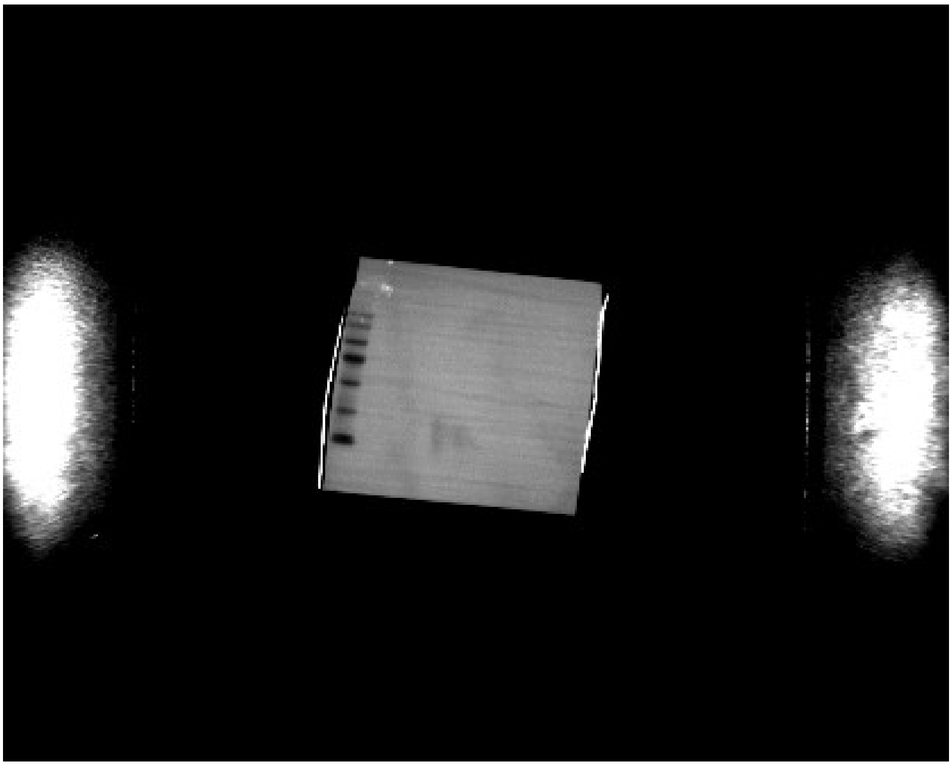

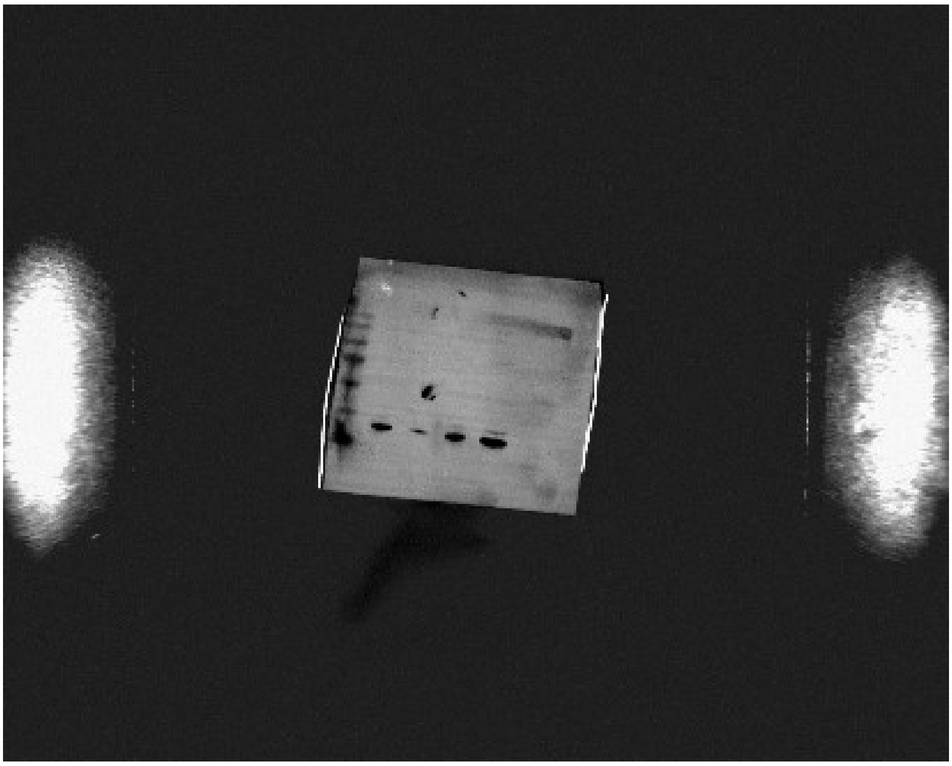

HIF-1a-2

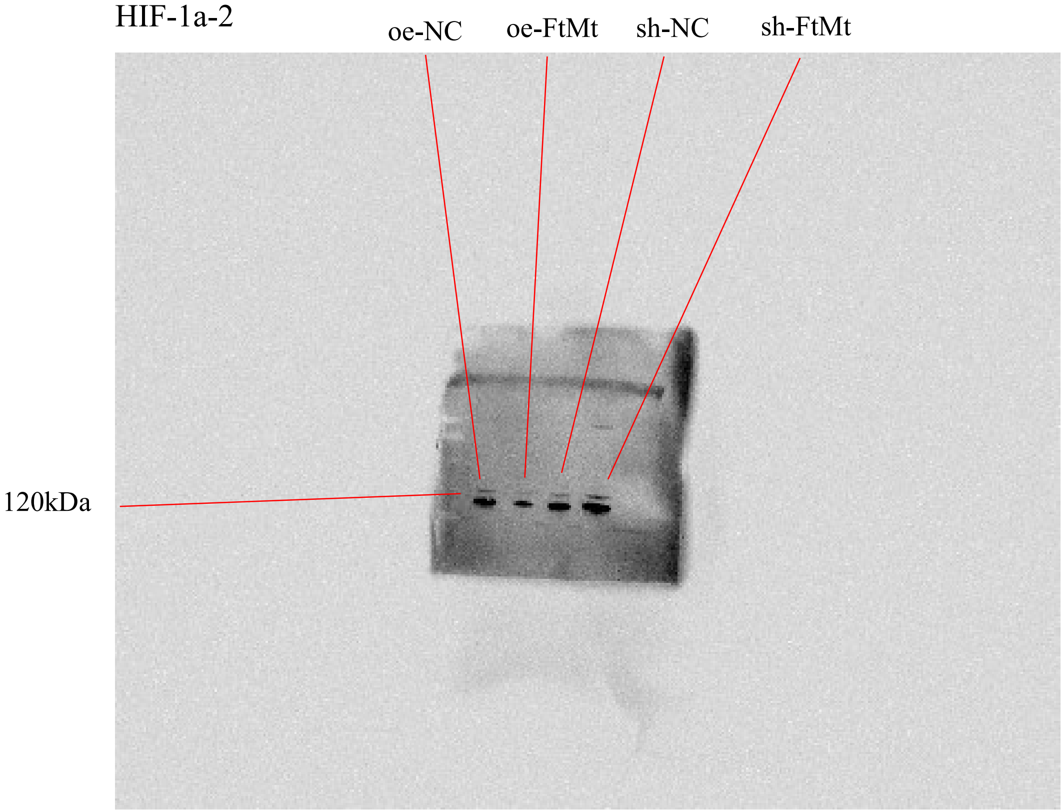

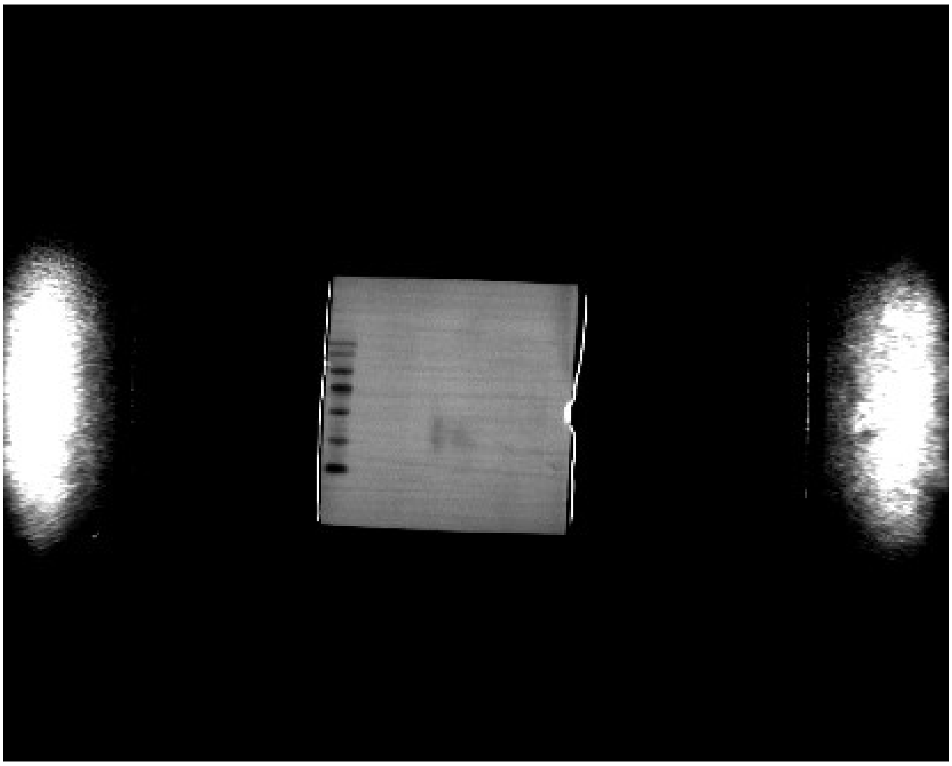

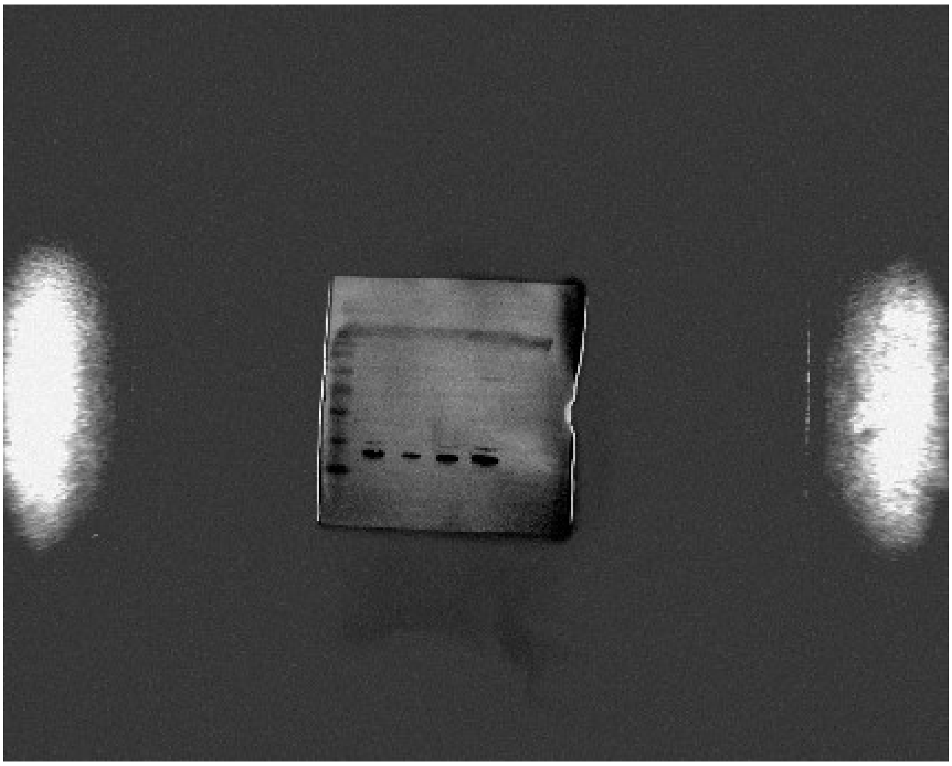

HIF-1a-3

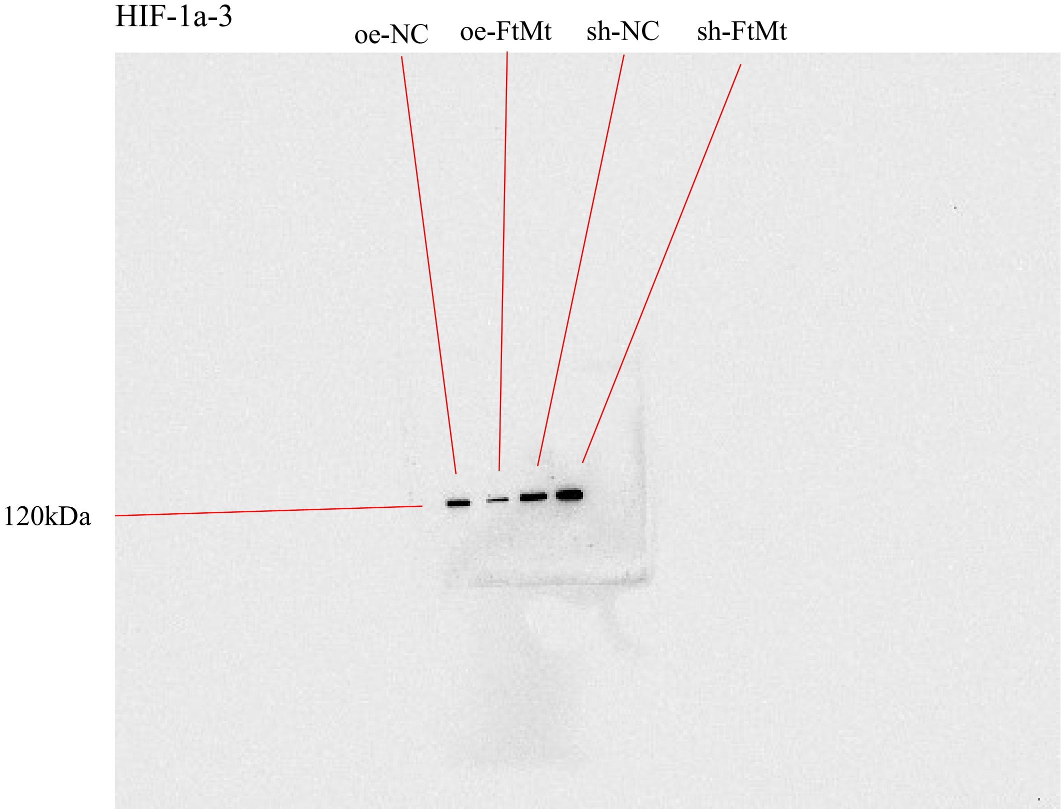

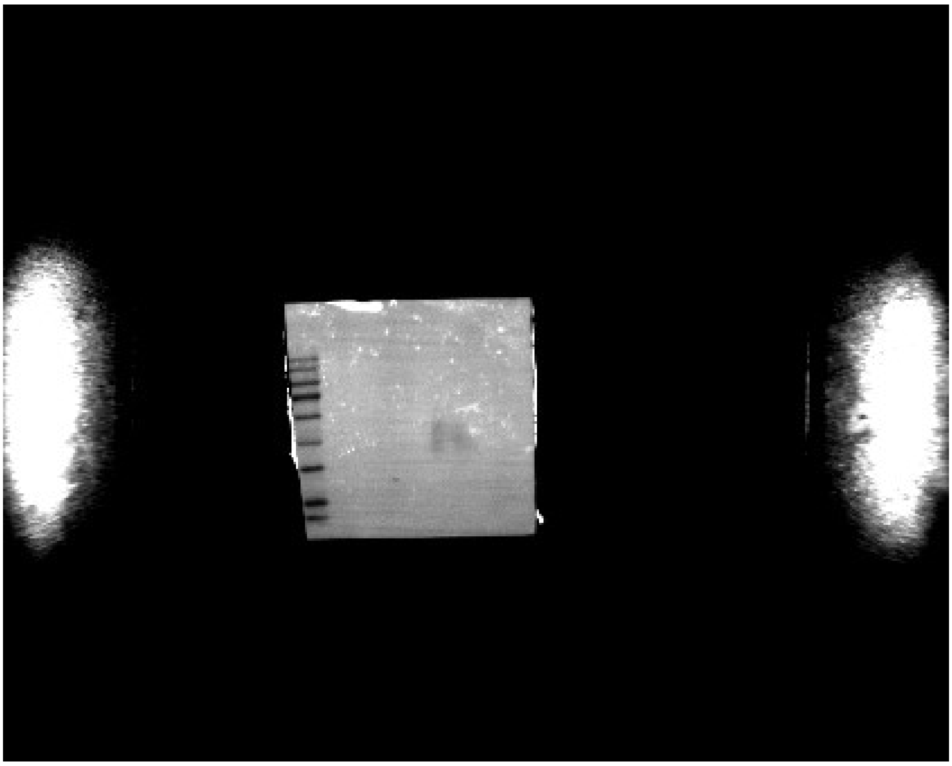

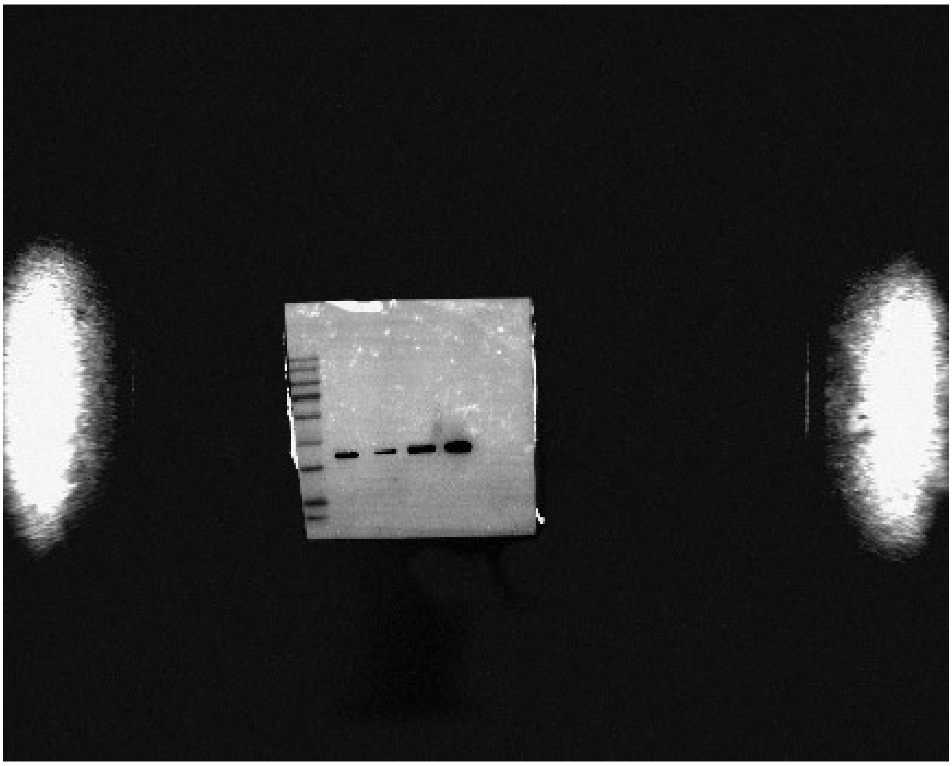

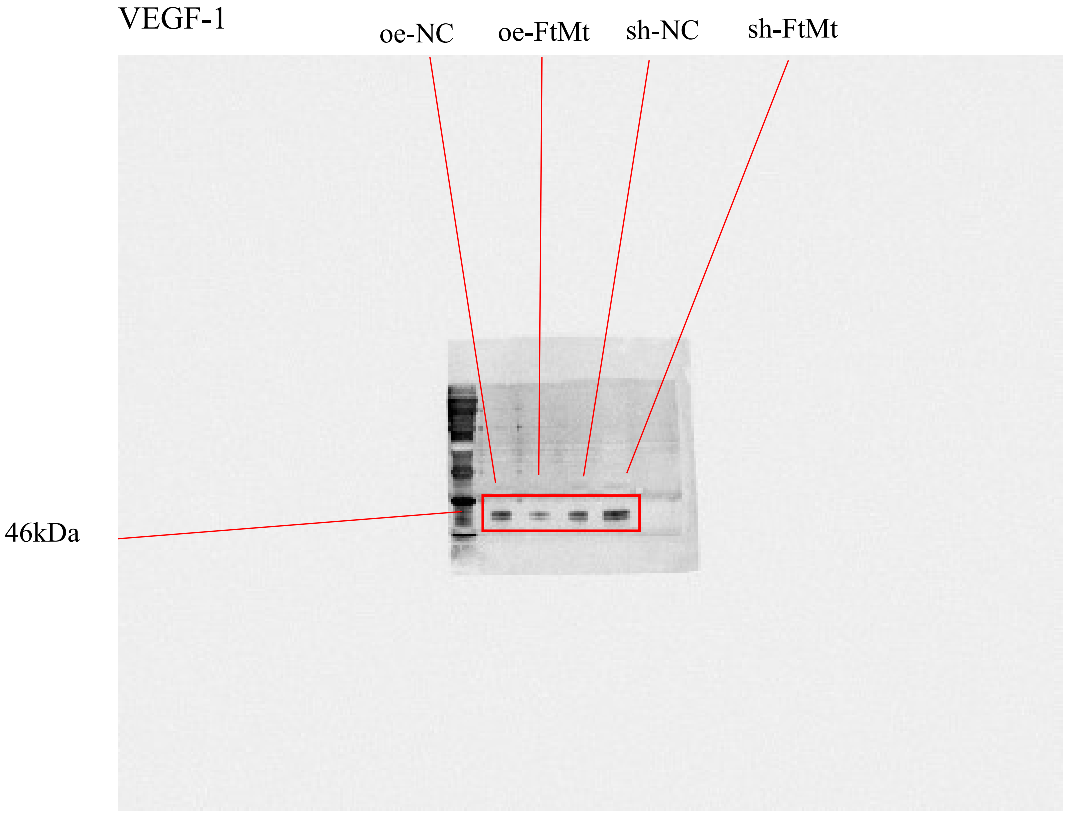

VEGF-1
oe-NC
oe-FtMt
sh-NC
sh-FtMt
46kDa

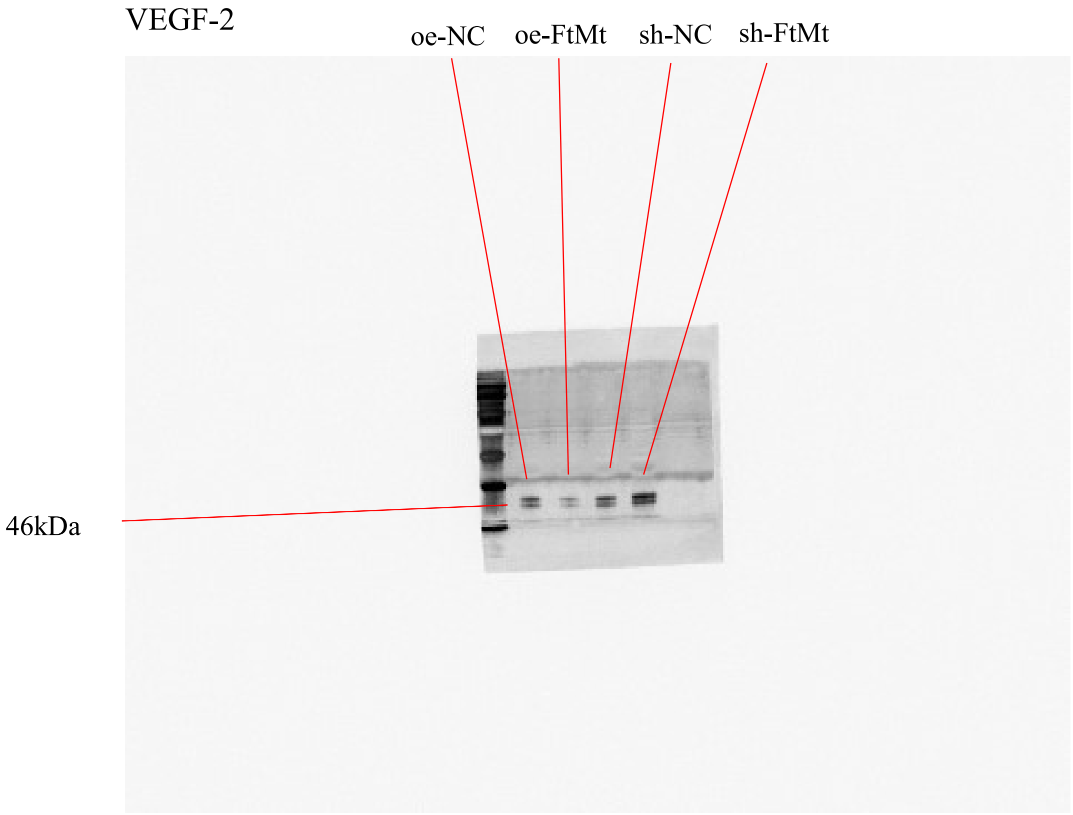

VEGF-2
oe-NC
oe-FtMt
sh-NC
sh-FtMt
46kDa

VEGF-3

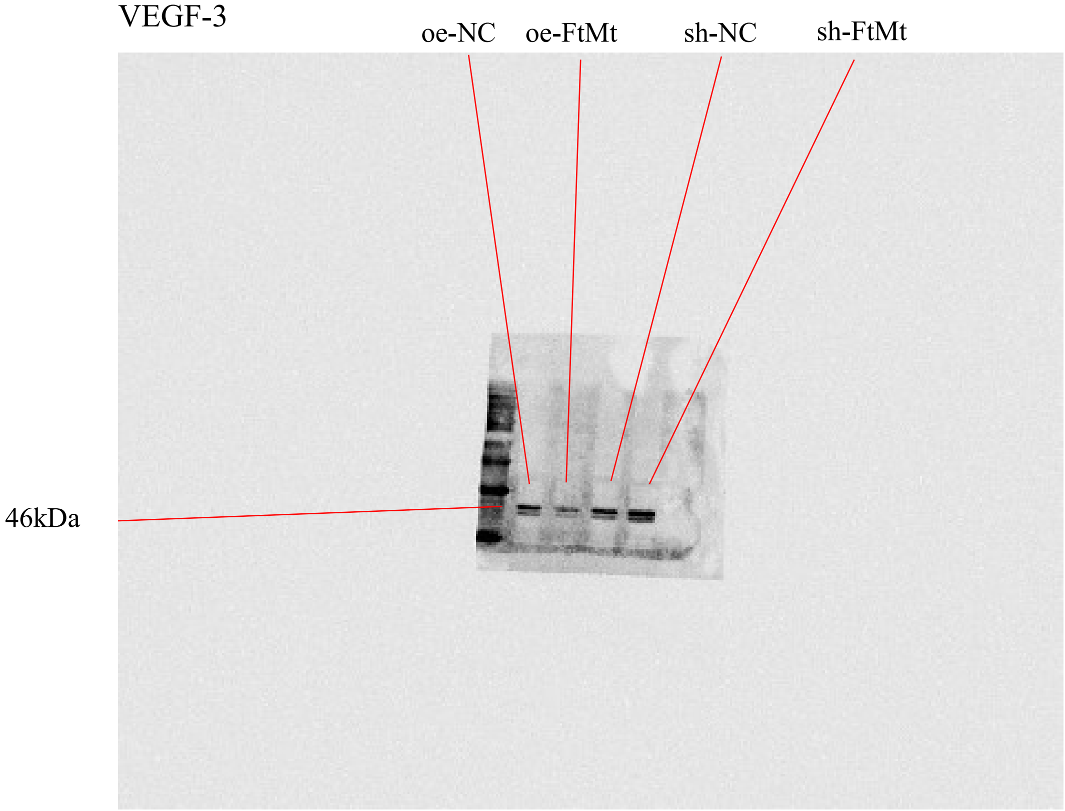

Supplement: Supplementary file 1 — Additional file 1. Supplementary file. [file 12884_2023_5448_MOESM1_ESM.pdf]
